# Severe head dysgenesis resulting from imbalance between anterior and posterior ontogenetic programs

**DOI:** 10.1038/s41419-019-2040-0

**Published:** 2019-10-24

**Authors:** Emmanuelle Grall, Victor Gourain, Asmaa Naïr, Elisabeth Martin, Marie-Christine Birling, Jean-Noël Freund, Isabelle Duluc

**Affiliations:** 1 Université de Strasbourg, Inserm, IRFAC/UMR-S1113, FMTS, 67200 Strasbourg, France; 2 Karlsruhe Institute of Technology, Institute of Toxicology and Genetics, 76021 Karlsruhe, Germany; 3 Institut Clinique de la Souris, 67404 Illkirch Cedex, France

**Keywords:** Disease model, Anatomy

## Abstract

Head dysgenesis is a major cause of fetal demise and craniofacial malformation. Although mutations in genes of the head ontogenetic program have been reported, many cases remain unexplained. Head dysgenesis has also been related to trisomy or amplification of the chromosomal region overlapping the *CDX2* homeobox gene, a master element of the trunk ontogenetic program. Hence, we investigated the repercussion on head morphogenesis of the imbalance between the head and trunk ontogenetic programs, by means of ectopic rostral expression of *CDX2* at gastrulation. This caused severe malformations affecting the forebrain and optic structures, and also the frontonasal process associated with defects in neural crest cells colonization. These malformations are the result of the downregulation of genes of the head program together with the abnormal induction of trunk program genes. Together, these data indicate that the imbalance between the anterior and posterior ontogenetic programs in embryos is a new possible cause of head dysgenesis during human development, linked to defects in setting up anterior neuroectodermal structures.

## Introduction

Head dysgenesis during embryonic development is a major cause of fetal demise occurring in ~1/250 conceptuses^1^. It covers a large spectrum of craniofacial and brain malformations and involves mutations in genes of the anterior ontogenetic program as well as environmental conditions. Yet, a great number of cases still remain unexplained^2^. During embryogenesis, the head is the first body part to form around gastrulation from resident anterior epiblast cells^3,4^. Unlike the head, the trunk is newly built by progressive posterior addition of tissue from the posterior epiblast through the primitive streak to get the three germ layers^5^. The posterior ontogenetic program notably differs from the head program by the involvement of an intricate network of homeobox transcription factor genes including the *caudal*-related *paraHox* genes *Cdx1/2/4*, the POU-homeobox gene *Oct4* and the genes of the four *Hox* clusters^6,7^. *Cdx1/2/4*, whose nested expression is limited rostrally in rhombomere 3, are crucial for maintaining the pool of posterior elongation progenitors, so that *Cdx*-null murine embryos fail developing body structures beyond the occiput^8^. *Oct4* sustains progenitor pluripotency^9^, and *Hox* genes^10^, together with *Cdx1/2*^11,12^, provide the growing posterior tissues their anteroposterior (AP) positional information. *Hox* clusters genes, limited anteriorly in rhombomere 2^13^, exhibit spatial and temporal colinearity^6,7^ under complex regulatory mechanisms involving notably the CDX factors^14,15^.

Among the three *CDX* paralogues, *CDX2*, located on human chromosome 13, is important not only for posterior elongation and patterning at gastrulation but also earlier in the trophectoderm^16,17^ and later in the gut^18,19^. Noteworthy, although *CDX2* is not normally expressed in head progenitors, head dysgenesis has been frequently associated with the trisomy of chromosome 13 (Patau syndrome)^20^ or with partial trisomy of the long arm of this chromosome including the region q12.2 that overlaps the *CDX2* locus^21,22^. The Patau syndrome is a rare and dramatic disease whose prevalence is estimated at 1:12,000 to 1:29,000 in newborns with a median survival time of 6–10 days^23^.

On this basis, the link between the amplification of the locus containing the posterior ontogenetic gene, *CDX2*, and the occurrence of rostral malformations led us to investigate the repercussion on head morphogenesis of disturbing the balance between anterior and posterior developmental programs, by means of ectopically expressing *CDX2* rostrally at gastrulation.

## Results

### Head dysgenesis caused by rostral ectopic expression of CDX2

**Fig. 1:**
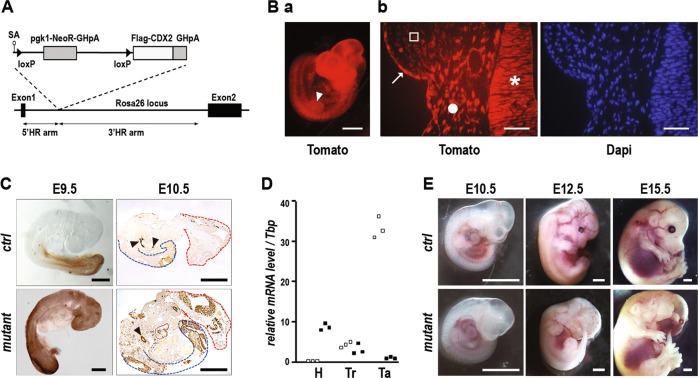
Morphologic alterations caused by rostral ectopic expression of CDX2. **A** The *RsCDX2* allele; SA: Splicing Acceptor site; GHpA: polyadenylation site of the human Growth Hormone gene. **Ba** Tomato fluorescence emitted by E10.5 *RosaCAG*^*tdTomato*^*::Sox2Cre*^*ERT2*^ embryos exposed to Tamoxifen at E6.5. The arrowhead points to the anterior limb bud. Bar: 500 µm. **b** Transversal sections in the telencephalon showing Tomato fluorescence emission at the level of the neuroepithelium (asterisk), neural crest derived cells (closed circle) and ectoderm (arrow) but not in the cephalic mesenchyme (open square). Bars: 50 µm. **C** Immunodetection of the CDX2 protein in whole-mount preparations of E9.5 control (ctrl) and *RsCDX2::Sox2Cre*^*ERT2*^ (mutant) littermates, and in sections of E10.5 control and mutant embryos. Red and blue dotted lines mark head and tail, respectively. Arrowheads show the endogenous Cdx2 in the gut endoderm. Bars: 500 µm. **D** Relative RNA levels by RTqPCR of the transcripts for endogenous *Cdx2* (open squares) and for the *CDX2* transgene (black squares) in the head (H), trunk (Tr) and tail (Ta) of 3 mutant embryos at E10.5. **E** Morphology of E10.5, E12.5 and E15.5 control and mutant embryos. Bars: 1 mm

Mice designed for inducible expression of the human *CDX2* homeobox gene, the *RsCDX2* mice, were generated by inserting into the *Ros26* locus the human *CDX2* cDNA preceded by a loxP-flanked transcriptional stop cassette (Fig. 1A). Ectopic expression of the CDX2 protein rostrally to its anterior limit in rhombomere 3 was achieved using these mice crossed with *Sox2Cre*^*ERT2*^ mice expressing Cre^ERT2^ in the whole epiblast at gastrulation, while the pregnant females received a single injection of Tamoxifen at day 6.5 *post-coitum*. The procedure was validated by demonstrating the rostral expression of Tomato protein in *RosaCAG*^*tdTomato*^*::Sox2Cre*^*ERT2*^ embryos (Fig. 1Ba), mainly at the level of the neuroepithelium, neural crest derived cells and ectoderm, but not in the cephalic mesenchyme (Fig. 1Bb). It was then successfully applied to *RsCDX2::Sox2Cre*^*ERT2*^ embryos to trigger ectopic expression of CDX2 in these tissues, as shown by whole-mount immunohistochemistry and immunostaining on tissue sections (Fig. 1C) and by RTqPCR (Fig. 1D). Although no macroscopic phenotype was displayed in mutants before E9.5, head dysmorphology characterized by a flattened anterior aspect appeared around E10.5, worsened at E12.5, and led to profound deformities at E15.5 (Fig. 1E; Supplementary Figure S1): the frontonasal process was missing leading to exencephaly, eyes were absent or limited to rudimentary structures and the maxillary branch of the first pharyngeal arch failed to merge axially. Half of the mutants also exhibited preaxial forelimb polydactyly (Supplementary Figure S1).

### Rostral ectopic expression of CDX2 perturbs the anterior ontogenetic program and induces elements of the posterior program

**Fig. 2:**
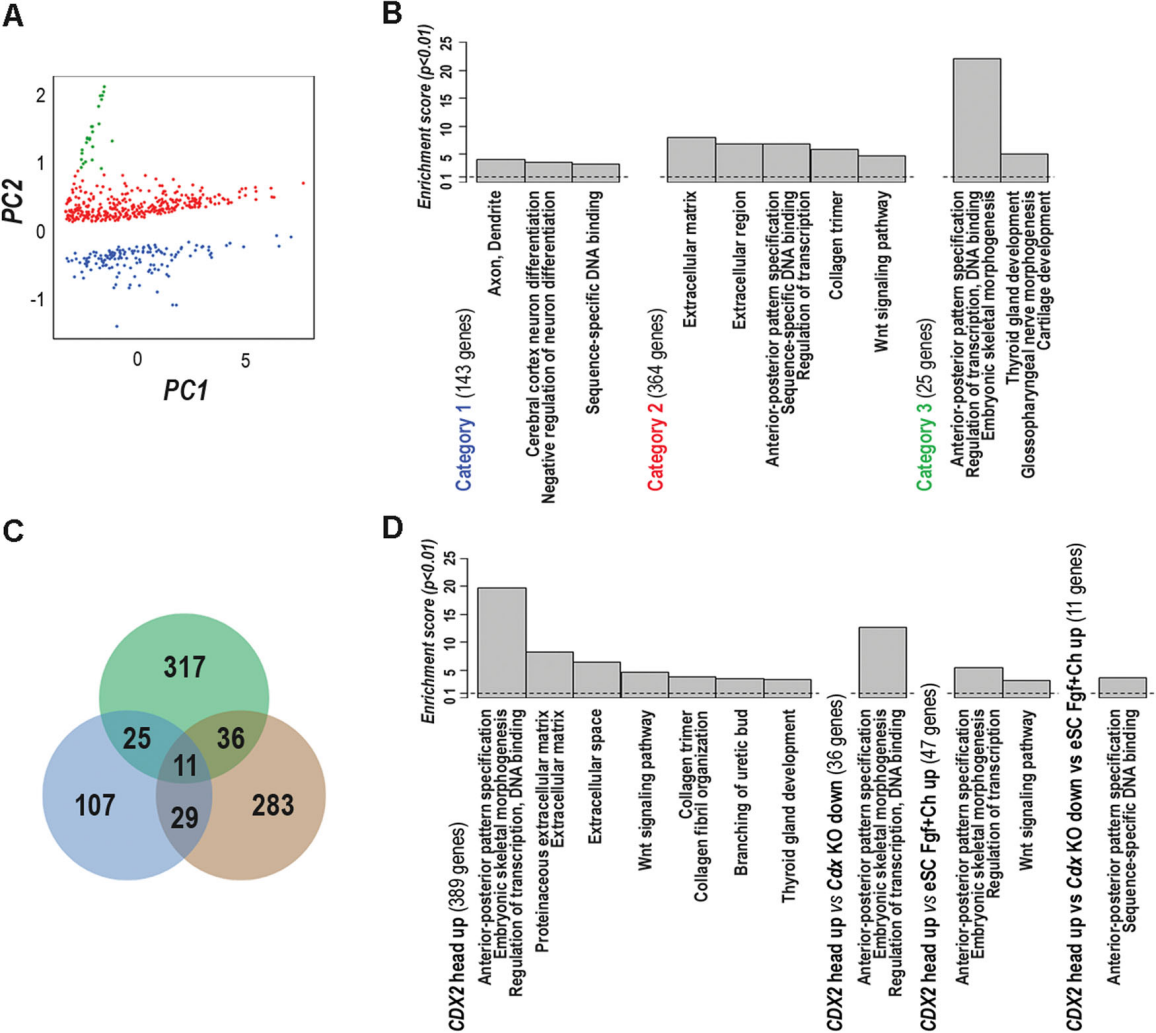
Gene modulation caused by rostral ectopic expression of CDX2. **A** Principal Component Analysis of the 532 differentially expressed genes by rostral ectopic expression of CDX2 in the head of E10.5 embryos (absolute Log2FC > 0.4, adjp < 0.05). **B** Functional Annotation Clustering (Enrichment score > 3; *p* < 0.01) of the 3 gene categories defined by Principal Component Analysis. pvalues were corrected with FDR multiple testing method. **C** Venn representation of the genes upregulated by ectopic expression of CDX2 in the head (green), of those upregulated in embryonic stem cells treated with CHIR99021 plus Fgf8 (brown), and of those downregulated in *Cdx*-null embryos (blue). **D** GO term representation (Enrichment score > 3; *p* < 0.01) for the lists of common genes displayed by the comparative analysis in **D**

Transcriptome analysis of the head of E10.5 control and mutant *RsCDX2::Sox2Cre*^*ERT2*^ littermates identified 532 differentially-expressed genes (Supplementary Table S1) falling into 3 categories by Principal Component Analysis (Fig. 2A, B; Supplementary Table S2 sheet 1). Category 1 corresponded to the 143 genes downregulated in mutants and functional annotation clustering revealed that it organized into 3 clusters respectively associated with the Gene Ontology (GO) terms Axon/Dendrite; Cerebral cortex neuron differentiation/Negative regulation of neuron differentiation; and Sequence-specific DNA binding. Several of the downregulated genes encoding DNA binding factors are crucial for head development like *Otx1* (brain and sensory organ development), *Foxg1*, *Nkx2.1* and *Six3* (telencephalon formation), *Tal2* (midbrain formation), *Six2* (growth of the cranial base), *Ascl1* and *Myb* (neuronal commitment and progenitor proliferation), *Nr2e1* (forebrain and retina stem cell determination), *Six3*, *Six6*, *Eya4*, *Vax1* and *Vsx2* (eye morphogenesis), *Tcf7L2* (radial migration of cortical neurons), *Fezf2* and *Bcl11a* (subcerebral neurons specification), *Helt* (GABAergic neurogenesis), *Rora* (Purkinje cells development), and *Zbtb20* (hippocampal neuronal determination). Thus, the head ontogenetic program is largely perturbed following the rostral induction of *CDX2* expression. Unlike the category 1, the categories 2 and 3 covered the 389 upregulated genes. Category 2 (364 genes) subdivides into 5 clusters associated with the GO terms Extracellular matrix region; Anterior–posterior patterning/Sequence-specific DNA binding/Regulation of transcription; Collagen; and Wnt signaling. Category 3 (25 genes) presents the cluster with the highest enrichment score, related to the GO terms Anterior-posterior patterning/Sequence-specific DNA binding/Regulation of transcription/Embryonic skeletal morphogenesis. To go further into the characterization of the 389 genes upregulated by rostral ectopic expression of *CDX2*, the list of genes of the categories 2 and 3 was compared with two other experimental conditions where the *Cdx* function is altered^14^: the genes downregulated in E8 *Cdx*-null embryos in which the trunk program is arrested, and the genes upregulated in epiblast stem cells cultured with a Wnt agonist plus Fgf8 to recapitulate the onset of the posterior program including *Cdx2* (Fig. 2C, D; Supplementary Table S2 sheet 2). Several genes arose by pairwise comparison while the 3 lists exhibited a set of 11 genes in common. Remarkably, the genes identified in the pairwise comparisons as well as the 11 genes in common in the 3 experimental conditions are related to the GO terms Anterior-posterior patterning/DNA binding/Regulation of transcription. This highlights that rostral *CDX2* induction not only perturbs the normal anterior ontogenetic program of the head, but it also turns on elements of the posterior program normally active in the developing trunk.

### Ectopic CDX2 turns on the expression of all central *Hox* clusters genes

**Fig. 3:**
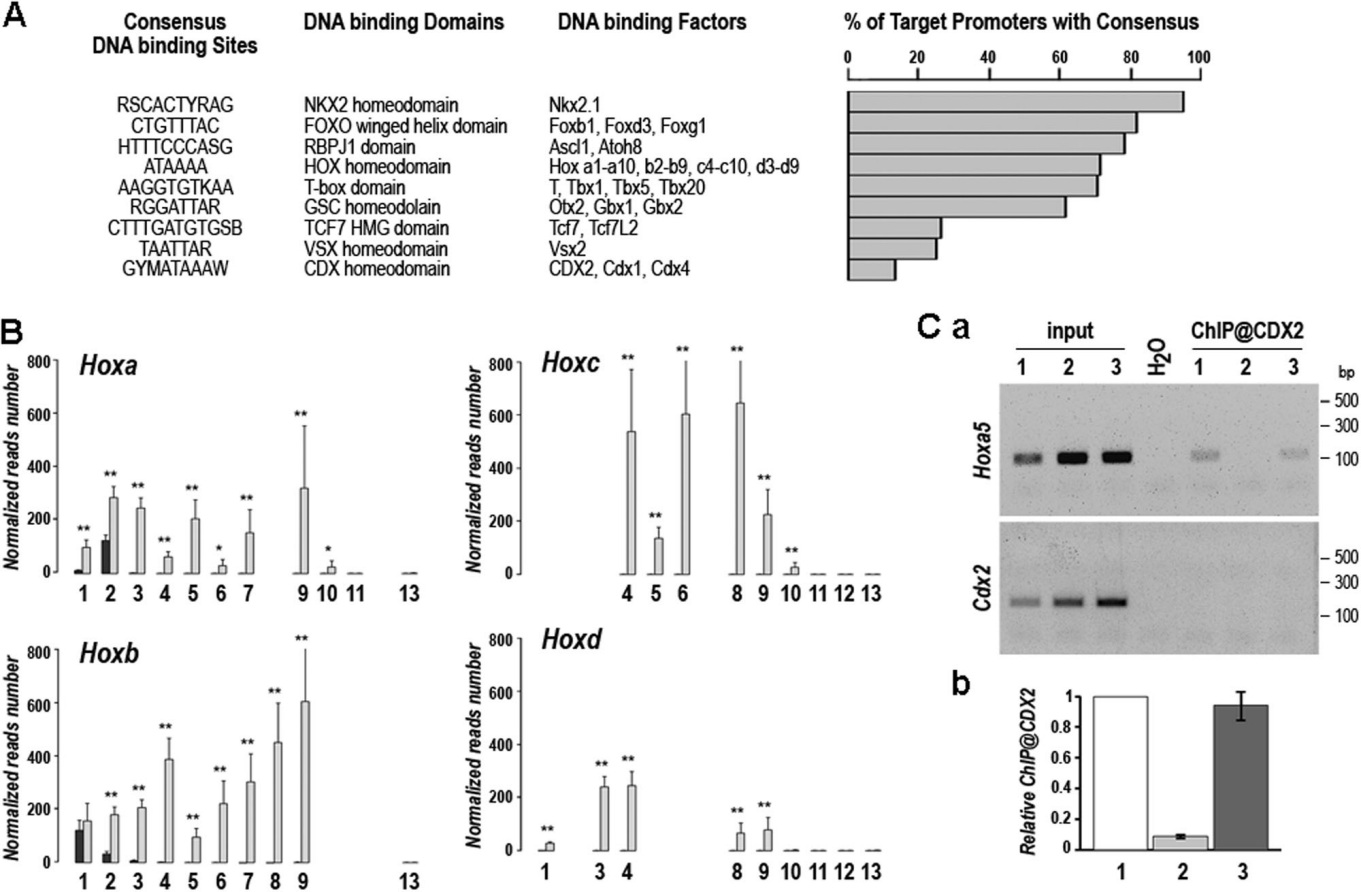
Homeobox gene changes caused by the ectopic expression of CDX2. **A** Enrichment analysis (*p* < 0.01) in DNA binding sites present in the −2000/ + 50-bp promoters of the 532 differentially-expressed genes. **B** Expression of the *Hox* clusters genes in control (black boxes) and mutant (gray boxes) E10.5 littermates. **Ca** CDX2 chromatin immunoprecipitation of *Hoxa5* promoter DNA in the trunk (lanes 1) and head (lanes 2) of an E10.5 control embryo and in the head of a mutant littermate (lanes 3). **b** Quantification by qPCR of the chromatin immunoprecipitation of *Hoxa5* promoter DNA in the trunk (lanes 1) and head (lanes 2) of an E10.5 control embryos and in the head of mutant littermates (lanes 3) (*n* = 3). The results are expressed relative to the value fixed at 1 in the trunk of the controls

Transcription factor genes represent 19.7% (105/532) of the genes deregulated by ectopic expression of *CDX2* (Supplementary Table S2 sheet 3). Their relevance is supported by the significant enrichment in DNA binding sites for 45 of them (42.9%; FDR < 0.02) in the −2000/ + 50-bp promoters of the whole set of deregulated genes (Fig. 3A). These 45 factors fall into 9 groups according to their DNA binding domain. Homeobox genes, known to be crucial in morphogenesis, represent 48 of the 105 deregulated transcription factor genes and correspond to 5 of the 9 groups identified by DNA binding sites enrichment. Importantly, these homeobox genes include the majority (24/39) of the genes of the four *Hox* clusters, especially those involved in trunk patterning (Fig. 3B). Indeed, while transcripts of the anterior genes *Hoxa1*, −*a2*, −*b1*, and −*b2* were already present in the head of control embryos, consistent with their normal anterior limit in the hindbrain, all the 17 central cluster genes from position 3 to 9-10, also belonging to the set of 25 genes of the category 3 in the Principal Component Analysis, were unexpressed in the head of control embryos but turned on in the mutants. More posterior *Hox* genes at positions 11–13, controlled by *Gdf11* instead of *Cdx* in the trunk-tail transition^24^, remained silent. Chromatin immunoprecipitation (ChIP) targeting the Cdx2-binding site previously identified and functionally characterized upstream of the *Hoxa5* gene^15,25^ showed occupancy by the endogenous Cdx2 protein in the trunk but not in the head of control E10.5 embryos, as expected; however, the CDX2 protein was actually bound to this site in the head of *RsCDX2::Sox2Cre*^*ERT2*^ littermates (Fig. 3C), demonstrating the direct interaction of CDX2 with the *Hoxa5* promoter when this gene becomes turned on in the head of mutant embryos. Thus, ectopic expression of *CDX2* is able to switch on central *Hox* genes in the head, similar to what happens during trunk morphogenesis.

### Ectopic CDX2 perturbs cell fate and cell interactions

**Fig. 4:**
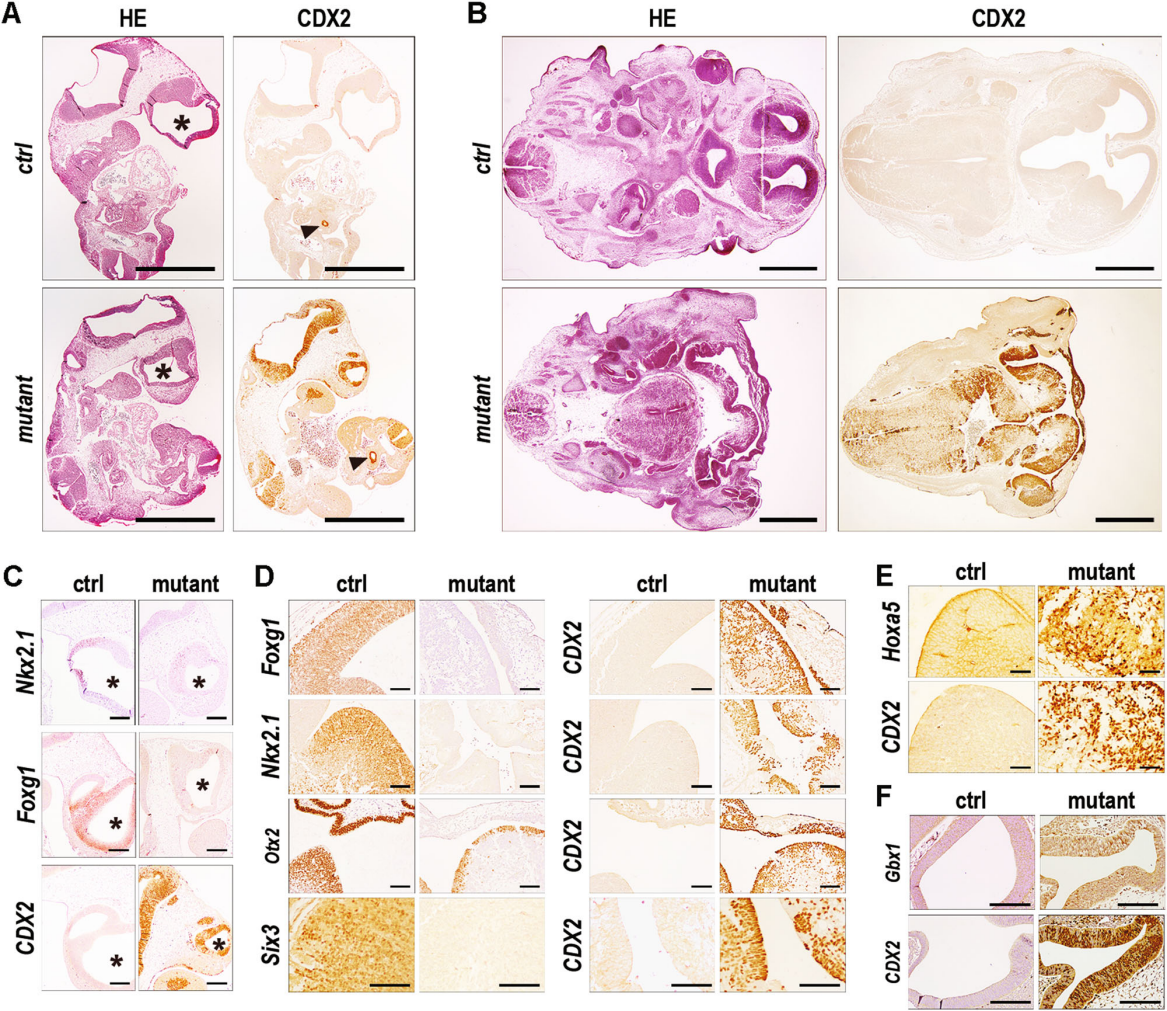
Histology and transcription factor alterations by rostral ectopic expression of CDX2. **A** Histology and CDX2 immunostaining in parasagittal sections of E10.5 control and mutant littermates. Asterisks show forebrain vesicles and arrowheads the endogenous Cdx2 protein in the gut endoderm. Bars: 1 mm. **B** Same as **A** for transversal head sections of E13.5 control and mutant littermates. Bars: 1 mm. **C** Transcription factor immunodetection in parasagittal head sections of E10.5 control and mutant littermates. Asterisks: telencephalic vesicles. Bars: 200 µm. **D** Same as **C** in transversal head sections of E13.5 control and mutant embryos. Bars: 100 µm. **E** Same as **D**. Bars: 100 µm. **F** Same as **C**. Bars: 50 µm

Histology was analyzed in mutants between E9.5 and E15.5 and representative pictures of embryos at day 10.5 and 13.5 are illustrated in Fig. 4A, B. As shown at E13.5, profound tissue disorganization occurred rostrally where ectopic CDX2 was the highest in the neuroepithelium. The shape of the diencephalon and third ventricle was altered together with the atrophied structures of the telencephalon and lateral ventricles; the choroid invagination was missing and eyes were absent or poorly formed. Craniofacial structures were also missing. These alterations connected with the reduced level or absence of forebrain transcription factors, as shown by immunohistochemistry at E10.5 for Nkx2.1 and Foxg1 in the telencephalon (Fig. 4) and even more pronounced at E13.5 for Foxg1 in the lateral ventricles, Nkx2.1 in the medial ganglionic eminence, Otx2 in the medial ganglionic eminence and choroid plexus and Six3 in the diencephalon (Fig. 4D). The decrease of these proteins was in line with the RNAseq data. Reciprocally, RNAseq had revealed the increase of mRNAs for transcription factors normally expressed in the trunk like *Hox* genes, and/or caudally to the forebrain in the mid/hindbrain, such as *Gbx1*, *Gbx2*, *En1*, *Foxb1*, *Foxd3* and *Hes3*. Actually, the Hox5 protein was detected in the atrophied lateral ventricles of E13.5 mutants, namely more rostrally than the normal anterior limit of *Hox* genes (Fig. 4E), and Gbx1 also was shifted rostrally in the telencephalon of E10.5 mutants compared to its normal expression caudal to hindbrain rhombomere 2 in control embryos (Fig. 4F).

**Fig. 5:**
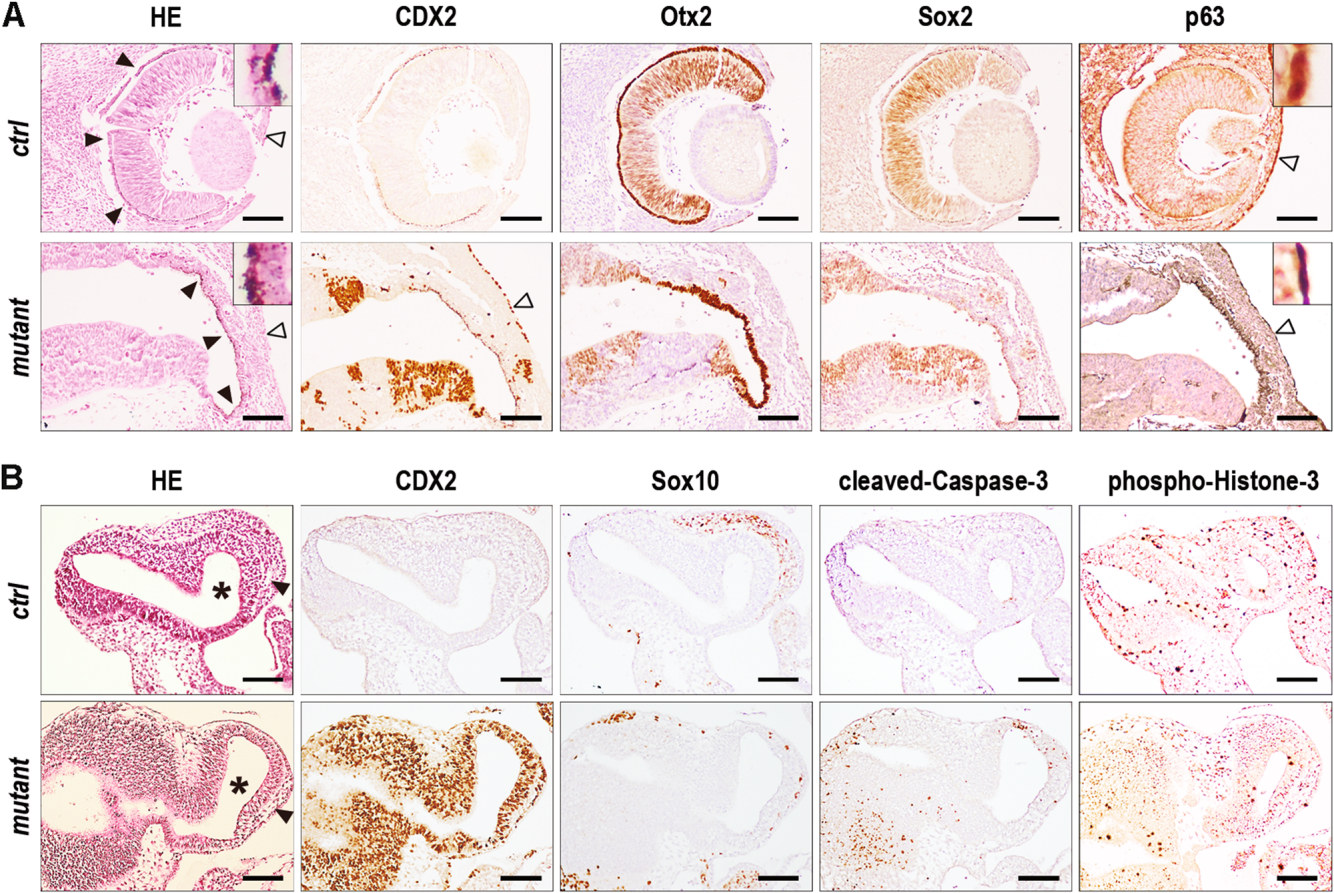
Eye and frontonasal malformations caused by rostral ectopic expression of CDX2. **A** Serial transversal sections of eyes and ocular remnants of E12.5 control and mutant littermates. Inserts are higher magnification of the retinal pigment epithelium and of the p63-positive ectodermal epithelium. Black and open arrowheads show retinal pigment cell layer and ectodermal layer, respectively. Bars: 100 µm. **B** Serial parasagittal sections of the frontonasal process (arrowheads) of E10.5 control and mutant littermates. Asterisks: telencephalic ventricles. Bars: 200 µm

Cell interactions also contribute to the morphological defects caused by ectopic CDX2, as exemplified in eye remnants (Fig. 5A). In a few E12.5 mutants, rudimentary ocular structures developed when the tip of the optic stalk remained CDX2-free. The neuroepithelium differentiated into a layer of Otx2-positive retinal pigment cells but failed to invaginate into optical cuff and to form the Sox2-positive neural layer. Concomitantly, the surface ectodermal layer facing the Otx2-positive neuroepithelium, also ectopically expressing CDX2, did not invaginate into the lens while maintaining p63 in contrast to its normal loss in the presumptive lens ectoderm^26^. Thus, ectopic CDX2 not only perturbs neuroepithelium development, but it also alters neuroepithelial-ectodermal interactions needed for eye morphogenesis.

**Fig. 6:**
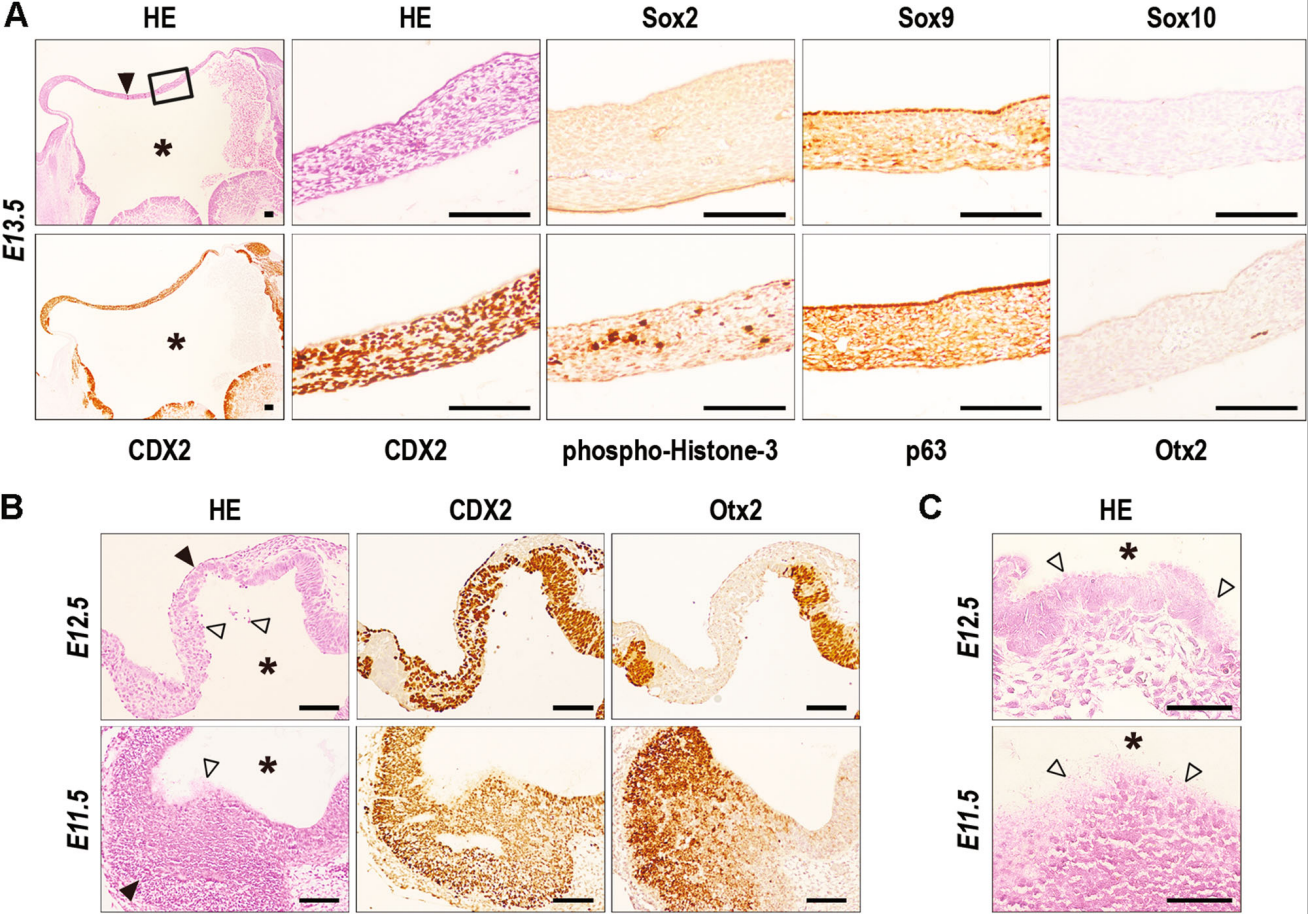
Progressive changes of the anterior-most wall of the forebrain by rostral ectopic expression of CDX2. **A** Serial transversal sections of the anterior-most wall (arrowheads) of the telencephalon (asterisks) in an E13.5 mutant embryo. Bars: 100 µm. **B** Serial transversal sections of an E12.5 mutant embryo and serial parasagittal sections of an E11.5 mutant embryo. Open arrowheads show cell delamination and cell debris from the neuroepithelium into the telencephalic ventricles. Bars: 100 µm. **C** Higher magnification of cell debris (open arrowheads) released in the telencephalic ventricles (asterisks) of E11.5 and E12.5 mutant embryos. Bars: 50 µm

Together with forebrain alterations, *RsCDX2::Sox2Cre*^*ERT2*^ mutants exhibited strong defects in the frontonasal process. The rostral shift in *Hox* expression corroborated the incompatibility of these genes with craniofacial development^27–29^. While the frontonasal process develops from the colonization of streams of diencephalic and anterior mesencephalic neural crest cells around E8.5-E9.5^3^, RNAseq data revealed mRNA changes for members of genes families involved in cell guidance during neural crest cell migration including Erbb3/4, Sema3b/6b, Nrp1/2 and Epha2/a3/a5/b1. Sox10 immunostaining used to label cells of neural crest origin showed a reduction of cell number in E10.5 mutants compared to controls, accompanied by cleaved-Caspase-3 staining and a low rate of phospho-Histone-3-positive cells (Fig. 5B). Thus, ectopic CDX2 compromises the migration and survival of neural crest cells intended to form the face. In addition, in absence of frontonasal process, the rostral-most wall of the forebrain was profoundly remodeled, as illustrated at E13.5 (Fig. 6A). The resulting structure was made of a compact, yet vascularized, cell mass covered by a single-layered CDX2-free but p63- and Sox9-positive epithelium. The cell mass strongly expressed CDX2, was proliferative, but devoid of all neuroepithelial factors tested here (Foxg1, Gbx1, Nkx2.1, Otx2, Six3, Sox2, Sox9) and also Sox10, suggesting no neural crest cell origin. Earlier stages at E11.5-E12.5 revealed the dynamics of the process where the Otx2-positive neuroepithelium expressing CDX2 became patchy and progressively replaced by CDX2-positive and Otx2-negative cells (Fig. 6B). This replacement was associated with the release of cellular material from the neuroepithelium into the telencephalic ventricles (Fig. 6C), without evidence of cleaved-Caspase-3 apoptosis. Thus, ectopic expression of CDX2 compromises the development of the facial neural crest-derived mesenchyme, associated with the disintegration of the neuroepithelium and its replacement by cells having lost neuroepithelial properties.

## Discussion

Although the impact of the loss of *CDX* function during embryonic development has been well documented, deciphering the primary role of this gene family in trunk elongation and patterning^8,30^, the effect of the abnormal anterior gain of function has not been studied so far in vertebrates. In *D. melanogaster*, forced expression of the *caudal* orthologue in the anterior pole at the syncytial embryonic stage induces head malformations, yet without homeotic changes^31^. Here, we developed the model of ectopic expression of *CDX2* in rostral tissues of mouse embryos to investigate the consequences of disturbing the balance between the anterior and posterior ontogenetic programs. The resulting embryos present with huge brain and craniofacial dysgenesis recapitulating several aspects of human malformations including holoprosencephaly. These malformations are associated with the decrease of important genes of the anterior program together with the rostral onset of elements of the posterior program among which are the trunk *Hox* genes. Unlike the endoderm, where ectopic CDX2 induces a homeotic shift from foregut to midgut-like properties^32^, in the rostral neuroepithelium this factor leads to missing head structures associated with perturbations in cell movement, proliferation and death, in particular at the level of neural crest cells and for the formation of eyes. This likely results from the interference between the endogenous anterior program of the head and the ectopic posterior program driven by the CDX2 protein known from gut studies to be a regulator of active chromatin organization^33,34^.

This work is highly relevant as regards head dysgenesis diseases in humans because a number of cases, among which the Patau syndrome, are associated with copy number amplification of the chromosome 13 locus overlapping *CDX2*. Thus, this study highlights that, in addition to single gene mutations in the head ontogenetic program^2^, the imbalance between head and trunk programs around gastrulation could be a novel basis of head dysgenesis. The mouse phenotype resulting from the forced expression of *CDX2* anteriorly above the normal anterior-posterior gradient, recapitulates observations made in human, including not only brain malformations, eye defects and frontonasal dysmorphology but also forelimb polydactyly. Together, these data open the way to further fetopathological studies to address in humans the importance of the imbalance between the anterior and posterior ontogenetic programs in head dysgenesis diseases of still unknown origin.

## Material and methods

### Mice and treatments

All the mouse experiments were performed in the certified animal facility (number H-67-482-21) according to the protocol approved by the French Ministry of Agriculture under the permit APAFiS#833.

The *RsCDX2* mouse was generated by inserting into the *Rosa26* locus the cassette made of a Splicing Acceptor site, an excisable fragment flanked by loxP sites and the FLAG-tagged form of the human CDX2 cDNA^35^. The excisable fragment contains the transcription unit formed by the *pgk1* promoter, the neomycin-resistance coding sequence and the Growth Hormone polyadenylation site. The *RsCDX2* mouse was generated at the Mouse Clinic Institute (Illkirch, France). A schematic representation of the *RsCDX2* allele is shown in Fig. 1Aa.

*Sox2Cre*^*ERT2*^ (stock number 017593)^36^ and *RosaCAG*^*tdTomato*^ (stock number 007909)^37^ mice were from the Jackson laboratory. Tail DNA and extraembryonic membrane DNA were respectively used for genotyping adult mice and embryos by PCR with the following primers: *RsCDX2* allele: GTGGTTTGTCCAAACTCATCA/CACGTGGTAACCGCCGTAGTC; *Sox2Cre*^*ERT2*^ allele: GCGGTCTGGCAGTAAAAACTATC/GTGAAACAGCATTGCTGTCACTT; *RosaCAG*^*tdTomato*^ allele: GGCATTAAAGCAGCGTATCC/CTGTTCCTGTACGGCATGG.

Pregnant females of *RsCDX2::Sox2Cre*^*ERT2*^ or *RosaCAG*^*tdTomato*^*::Sox2Cre*^*ERT2*^ intercrosses were given a single intraperitoneal injection of 2 mg of Tamoxifen in corn oil at day 6 after vaginal plug detection (E6.5 *post coitum*).

In all the figures illustrated for this work, control (*ctrl*) and mutant (*RsCDX2::Sox2Cre*^*ERT2*^) embryos were littermates; the controls were either wild-type or *RsCDX2* or *Sox2Cre*^*ERT2*^ embryos, giving identical results. Littermates with no detectable head expression of ectopic CDX2 protein by immunohistochemistry were excluded. For each developmental stage, embryos from 3 littermates with ectopic CDX2 expression were analyzed. No randomization was used. Phenotypic analyses were performed in blind.

### Histology and immunohistochemistry

Whole-mount immunohistochemistry was performed on E9.5 embryos as described^38^. For this purpose, embryos were fixed overnight in DMSO/methanol (1:4), rehydrated in PBS, blocked in 2% dry milk, 0.5% Triton X100 and incubated overnight with rabbit anti-CDX2 antibody CDX2 (Abcam; ab76541; RRID:AB_1523334, dilution 1:5000). HRP-labeled goat anti-rabbit antibody (Vector Laboratories; PI-1000; RRID: AB_2336198, dilution 1:500) was added overnight and revealed with DAB. Embryos were cleared with Methyl Salicylate before observation.

For immunohistochemistry on tissue sections, E10.5 to E15.5 embryos were fixed with 4% PFA, embedded in paraffin and sectioned (5 µm) before staining with hematoxylin-eosin (HE) or by immunohistochemistry, as described^39^. Antibodies were: rabbit anti-cleaved-Caspase-3 (R&D System; AF835; RRID:AB_2243952, dilution 1:500), rabbit anti-CDX2 (Abcam; ab76541; RRID:AB_1523334, dilution 1:10,000), rabbit anti-Foxg1 (Abcam; ab18259; RRID:AB_732415, dilution 1:1000), rabbit anti-Gbx1 (dilution 1:5000)^40^, mouse anti-Hoxa5 (Santa Cruz; sc-515309, dilution 1:250), mouse anti-Nkx2.1 (Thermo Fischer; MA5-13961; RRID:AB_10984070, dilution 1:500), rabbit anti-Otx2 (Abcam; ab183951, dilution 1:250), rabbit anti-p63 (Abcam; ab124762; RRID:AB_10971840, dilution 1:2000), rabbit anti-phospho-Histone-3 (Millipore; 06-570; RRID:AB_310177, dilution 1:1000), guinea pig anti-Six3 (Rockland; 200-201-A26S; RRID:AB_1961548, dilution 1:1000), mouse anti-Sox2 (R&D System; MAB2018; RRID:AB_358009, dilution 1:500), rabbit anti-Sox9 (dilution 1:500)^41^, rabbit anti-Sox10 (Sigma-Aldrich; Ab383R, dilution 1:350). Secondary biotinylated antibodies were from Vector Laboratories and used at the dilution 1:200: goat anti-guinea pig (BA-7000; RRID:AB_2336132), goat anti-rabbit (BA-1000; RRID: AB_2313606), rabbit anti-goat (BA-5000; AB_2336126), goat anti-mouse (BA-9200; RRID:AB_2336171).

### RNA extraction, RTqPCR and RNAseq analyses

RNA was individually prepared using TRI Reagent (Euromedex, France) from each of 3 control and 3 mutant E10.5 littermate embryos sectioned to separate the head (sectioned under the binocular loop between the maxillary and mandibular components of the pharyngeal arch 1), the trunk and the tail. Total RNA was treated with DNAse (RQ1 RNAse-free DNAse, Promega Inc.) and its quality was assessed with a Bioanalyzer (Agilent).

For RTqPCR, reverse transcription was performed with 2 µg RNA using SuperScript II Reverse Transcriptase (Invitrogen) and Oligo(dT)12-18 primer (Invitrogen). Quantification of the endogenous *Cdx2* transcript and of the transcript of the *CDX2* transgene was performed using the TaqMan Master Mix and gene-specific TaqMan primers sets (TaqMan Gene Expression assays, Life Technologies Applied Biosystems): mouse *Cdx2*, Mm01212280_m1; human CDX2, Hs00230919_m1, and mouse Tbp, Mm00446973_m1. Analysis of the results obtained in triplicate was performed with the 7500 software v2.0.1 (Life Technologies Applied Biosystems) using the relative ΔΔCt quantification method.

For RNAseq, 1 µg of total RNA of each preparation made from the head of 3 control and 3 mutant embryos was used to prepare the sequencing libraries using the Illumina TruSeq stranded mRNA kit, following the supplier’s recommendations. The quality and the quantity of the libraries were both assessed on a High Sensitivity chip with a Bioanalyzer. The libraries were diluted to 10 pM to generate clusters in both lanes of a Rapid flowcell (Illumina). Sequencing was performed with a HiSeq1500 (Illumina) in paired-end for a read length of 50b. Cluster mapping was done with the software RTA (v1.13 Illumina). The base calling and the demultiplexing were performed with the software bcl2fastq (v2.17.1.14 Illumina).

The quality of the sequencing reads was assessed with FASTX-toolkit (v0.0.13) and no pre-processing was needed. The alignment of the reads was done with STAR v2.5^42^ against the reference genome of *Mus musculus* (GRCm38.90). To assess its expression, the human transgene hCDX2 (ENSG00000165556) was added to the reference murine genome as an additional gene and processed as the canonical ones. The aligner STAR was used with default parameters and the alignment was refined with a second pass. The read counting was done with HTSeq v0.6.1^43^ in union mode and at gene level. The normalization of gene expression as well as the consistency among biological replicates were both assessed with the R package DESeq2 1.10.1^44^. Differential expression analysis was also done with DESeq2. Prior to the Wald test, the values of log2(Fold Change) were not shrunk. The significantly differentially-expressed genes were identified based on the p-value, corrected with the False Discovery Rate (FDR) multiple testing method, and the log2(Fold Change). Genes with an absolute log2(Fold Change) ≥ 0.4 and an adjusted p-value < 0.05 were considered as differentially expressed. All the downstream analyses were done with R and with DAVID^45^; Gene Ontology enrichment was done with the one-tailed exact Fisher test and the pvalues were corrected with FDR multiple testing method.

The transcription factor binding motifs in the promoters (from 2000-bp upstream to 50-bp downstream of the transcription starting site) of the differentially expressed genes were analyzed with the software HOMER v4.10^46^. Only the known and curated motifs were used for the enrichment analysis. The enriched motifs were manually curated comparing with the most up-to-date version of JASPAR^47^ and Tomtom from the MEME software suite^48^.

### Chromatin immunoprecipitation

E10.5 embryos were harvested and sectioned under the binocular loop between the maxillary and mandibular components of the pharyngeal arch 1, and above the posterior limbs, to collect and freeze separately in liquid nitrogen the head and the trunk segments, respectively. Extraembryonic membranes were used for genotyping the embryos.

Chromatin immunoprecipitation (ChIP) assay was performed on the head and trunk segments of single control and mutant littermate embryos, using the ChIP IT High Sensitivity Kit (Active Motif), accordingly to the supplier’s recommendations. Volumes and durations of reactions were adapted to the weak amount of tissue recovered at this stage (5-20 mg tissue per sample). Briefly, embryonic tissues were fixed in 1% PFA then sonicated. Chromatin was incubated overnight with 2 µg of rabbit anti-CDX2 (Abcam; ab76541; RRID:AB_1523334). The antibody-bound protein/DNA complexes were precipitated using protein G agarose beads. After DNA purification on the DNA purification column, PCR reactions were performed on input and immunoprecipitated DNA for 38 cycles using primers flanking the Cdx2-binding site within the *HoxA5* promoter: CGCTTTAACCCCTTTCAATTAC/GCTAGGCCCAACCCTGTTAG^15^, or as negative control with primers corresponding to a genomic segment of the second intron of the *Cdx2* gene, devoid of Cdx2 binding site: TGGGGCAATCTTAATGGGTA/TGTAGCCTTGACTTGGCTTT^19^. Quantification of the ChIP was performed by Sybr Green qPCR with the above *HoxA5* primers and the FastStart Universal SYBR Green Master kit (Roche), using the ΔΔCt method relative to the Input and fixed at 1 for the value obtained in the samples of the trunk.

## Supplementary information

Table S1

Table S2

Supplementary Figure Legend

Supplementary Fig S1

Declaration of contributions to article

## Data Availability

RNAseq data are deposited in the GEO database under the accession number GSE123559.
