# Supplementary material for: Severe head dysgenesis resulting from imbalance between anterior and posterior ontogenetic programs
Source: Cell Death Dis. 2019 Oct 24;10(11):812. doi: 10.1038/s41419-019-2040-0 (PMC6813351; doi:10.1038/s41419-019-2040-0)
Supplement: Supplementary file 1 — Table S1 [file 41419_2019_2040_MOESM1_ESM.docx]

**Table S1**

Differentially-expressed genes in the head of *CDX2*-expressing *vs* control E10.5 littermates.

| **ensGeneID** | **Gene Symbol** | **log2FoldChange** | **Fold Change** | **adjp** |
| --- | --- | --- | --- | --- |
| ENSMUSG00000032300 | 1700017B05Rik | 0,463109769 | 1,378510034 | 0,015338344 |
| ENSMUSG00000043773 | 1700048O20Rik | -0,442888926 | 0,73566001 | 0,038325435 |
| ENSMUSG00000086841 | 2410006H16Rik | 0,516171258 | 1,430154746 | 0,014341609 |
| ENSMUSG00000074415 | 2610203C20Rik | -0,513911923 | 0,700320914 | 0,002982645 |
| ENSMUSG00000096001 | 2610528A11Rik | 6,334365051 | 80,69263249 | 3,23217E-05 |
| ENSMUSG00000058833 | 2810428I15Rik | 0,572518611 | 1,487117468 | 0,030323215 |
| ENSMUSG00000021098 | 4930447C04Rik | -0,788099047 | 0,579106644 | 2,49199E-05 |
| ENSMUSG00000041605 | 5730559C18Rik | 0,446935469 | 1,363141633 | 0,040253539 |
| ENSMUSG00000056468 | 5730596B20Rik | 8,57311084 | 380,8583828 | 1,63701E-06 |
| ENSMUSG00000102995 | A330074H02Rik | -1,517995862 | 0,349170635 | 0,005269947 |
| ENSMUSG00000025194 | Abcc2 | 2,270293108 | 4,824211331 | 0,032141934 |
| ENSMUSG00000003346 | Abhd17a | 0,471102795 | 1,38616865 | 0,002333645 |
| ENSMUSG00000007950 | Abhd8 | 0,486470893 | 1,401013534 | 0,044769845 |
| ENSMUSG00000095041 | AC149090.1 | -0,492605952 | 0,710740122 | 0,009631176 |
| ENSMUSG00000112343 | AC154734.1 | -1,675734246 | 0,313006767 | 1,67214E-12 |
| ENSMUSG00000020681 | Ace | 1,411391618 | 2,659936155 | 0,002024355 |
| ENSMUSG00000062825 | Actg1 | 0,40110596 | 1,320519824 | 1,49266E-05 |
| ENSMUSG00000025964 | Adam23 | -0,514304323 | 0,700130459 | 0,00303584 |
| ENSMUSG00000049538 | Adamts16 | 1,290120772 | 2,445485265 | 0,008339892 |
| ENSMUSG00000066113 | Adamtsl1 | 1,24969443 | 2,377910524 | 0,010654403 |
| ENSMUSG00000037692 | Ahdc1 | 0,692972193 | 1,616610578 | 0,006830093 |
| ENSMUSG00000109311 | AI314278 | -1,601167253 | 0,329610189 | 0,045306239 |
| ENSMUSG00000011096 | Akt1s1 | 0,568557048 | 1,483039525 | 0,029742518 |
| ENSMUSG00000053279 | Aldh1a1 | -2,034304548 | 0,244125593 | 2,03096E-09 |
| ENSMUSG00000013584 | Aldh1a2 | 3,633179005 | 12,40783073 | 2,40659E-28 |
| ENSMUSG00000024747 | Aldh1a7 | -1,256752098 | 0,418485024 | 3,43029E-05 |
| ENSMUSG00000035561 | Aldh1b1 | 1,179480234 | 2,26495162 | 1,20999E-11 |
| ENSMUSG00000032531 | Amotl2 | 0,486023946 | 1,400579568 | 2,97206E-05 |
| ENSMUSG00000021314 | Amph | 0,40370031 | 1,322896607 | 0,049473323 |
| ENSMUSG00000032826 | Ank2 | -0,417867319 | 0,74853033 | 0,004767086 |
| ENSMUSG00000031075 | Ano1 | -0,945149352 | 0,519375781 | 3,60973E-08 |
| ENSMUSG00000039062 | Anpep | 1,088298035 | 2,126230545 | 0,000931329 |
| ENSMUSG00000004931 | Apba3 | 0,54554464 | 1,459571251 | 0,013237543 |
| ENSMUSG00000079042 | Apela | 2,555721744 | 5,879615221 | 8,73722E-09 |
| ENSMUSG00000037010 | Apln | 0,503485759 | 1,417634637 | 0,032933087 |
| ENSMUSG00000057315 | Arhgap24 | 1,278466332 | 2,425809622 | 0,000735477 |
| ENSMUSG00000035697 | Arhgap45 | 0,442878581 | 1,359313836 | 0,00416844 |
| ENSMUSG00000004661 | Arid3b | 0,422636099 | 1,340374457 | 0,013871909 |
| ENSMUSG00000029622 | Arpc1b | 0,516507837 | 1,430488438 | 2,91095E-05 |
| ENSMUSG00000020052 | Ascl1 | -0,732478709 | 0,601868948 | 0,021187512 |
| ENSMUSG00000076441 | Ass1 | 0,907131332 | 1,875312899 | 0,001236532 |
| ENSMUSG00000037621 | Atoh8 | 0,435214551 | 1,352111898 | 0,025727664 |
| ENSMUSG00000097428 | AW047730 | -1,690830926 | 0,309748473 | 0,000144042 |
| ENSMUSG00000000142 | Axin2 | 0,40411248 | 1,323274605 | 0,011235647 |
| ENSMUSG00000006731 | B4galnt1 | 0,882613435 | 1,843712153 | 0,011198079 |
| ENSMUSG00000030847 | Bag3 | 0,441267256 | 1,357796485 | 0,00303584 |
| ENSMUSG00000002980 | Bcam | 0,643297874 | 1,561895437 | 1,96659E-05 |
| ENSMUSG00000000861 | Bcl11a | -0,867286381 | 0,548176968 | 0,040686599 |
| ENSMUSG00000040093 | Bmf | 0,437762841 | 1,354502298 | 7,78483E-05 |
| ENSMUSG00000015943 | Bola1 | 0,871448484 | 1,829498819 | 0,001672141 |
| ENSMUSG00000114133 | BTF3L4 | -1,547798752 | 0,342031534 | 0,013877223 |
| ENSMUSG00000062638 | Btnl1 | 3,131703777 | 8,764694327 | 0,000591917 |
| ENSMUSG00000024340 | Btnl2 | 2,659838868 | 6,319624628 | 0,025248721 |
| ENSMUSG00000073420 | Btnl5-ps | 3,661661611 | 12,65522814 | 0,002906755 |
| ENSMUSG00000058914 | C1qtnf3 | 2,388043379 | 5,234469675 | 6,57294E-06 |
| ENSMUSG00000004110 | Cacna1e | -0,730255648 | 0,602797088 | 0,010861148 |
| ENSMUSG00000034889 | Cactin | 0,497214412 | 1,411485602 | 0,019482484 |
| ENSMUSG00000032936 | Camkv | 0,428300486 | 1,345647453 | 0,007365761 |
| ENSMUSG00000070372 | Capza1 | 0,57869354 | 1,493496172 | 0,014715268 |
| ENSMUSG00000000805 | Car4 | 1,409725842 | 2,65686669 | 1,77527E-08 |
| ENSMUSG00000033170 | Card10 | 0,667074672 | 1,587850045 | 0,002333645 |
| ENSMUSG00000063605 | Ccdc102a | 0,519876637 | 1,433832638 | 0,009805751 |
| ENSMUSG00000095098 | Ccdc85b | 1,257781382 | 2,391277204 | 0,000794633 |
| ENSMUSG00000049521 | Cdc42ep1 | 0,490539223 | 1,404969901 | 0,003291299 |
| ENSMUSG00000050840 | Cdh20 | -0,636478222 | 0,643281355 | 0,04499338 |
| ENSMUSG00000026312 | Cdh7 | -0,755330681 | 0,592410583 | 0,023762689 |
| ENSMUSG00000036510 | Cdh8 | -1,401652641 | 0,378495318 | 4,42152E-07 |
| ENSMUSG00000025497 | Cdhr5 | 2,862223112 | 7,271349355 | 7,47942E-07 |
| ENSMUSG00000026437 | Cdk18 | 0,580939442 | 1,495822969 | 0,03609265 |
| ENSMUSG00000023067 | Cdkn1a | 0,445180182 | 1,361484146 | 0,02216969 |
| ENSMUSG00000006585 | Cdt1 | 0,54094515 | 1,454925368 | 0,001406602 |
| ENSMUSG00000024619 | Cdx1 | 2,717953829 | 6,579389983 | 2,96688E-06 |
| ENSG00000165556 | CDX2(human) | 12,45754873 | 5624,654366 | 0,000289492 |
| ENSMUSG00000031326 | Cdx4 | 12,21085689 | 4740,609664 | 0,001587941 |
| ENSMUSG00000074272 | Ceacam1 | 1,328538595 | 2,511481409 | 0,019659587 |
| ENSMUSG00000002107 | Celf2 | -0,479222023 | 0,71736436 | 0,000539227 |
| ENSMUSG00000030077 | Chl1 | 2,495736949 | 5,640163361 | 7,32408E-11 |
| ENSMUSG00000032997 | Chpf | 0,716973396 | 1,643730064 | 0,000369359 |
| ENSMUSG00000068547 | Clca4a | 2,500869431 | 5,660264343 | 0,005747278 |
| ENSMUSG00000070473 | Cldn3 | 0,990620303 | 1,987039158 | 0,045782643 |
| ENSMUSG00000023959 | Clic5 | 1,462759914 | 2,75635157 | 0,004116371 |
| ENSMUSG00000044288 | Cnr1 | -0,598673913 | 0,660360662 | 0,012351839 |
| ENSMUSG00000030092 | Cntn6 | -1,006463854 | 0,49776481 | 0,001161676 |
| ENSMUSG00000024330 | Col11a2 | 0,809192758 | 1,75223073 | 9,81482E-08 |
| ENSMUSG00000022371 | Col14a1 | 2,982051615 | 7,901089563 | 4,36278E-14 |
| ENSMUSG00000040690 | Col16a1 | 0,621777314 | 1,538769686 | 0,004812811 |
| ENSMUSG00000001506 | Col1a1 | 0,694543376 | 1,618372125 | 0,012960537 |
| ENSMUSG00000031274 | Col4a5 | 0,575681021 | 1,490380828 | 0,000688727 |
| ENSMUSG00000031273 | Col4a6 | 0,989224986 | 1,985118301 | 3,06766E-05 |
| ENSMUSG00000026837 | Col5a1 | 0,627637431 | 1,545032763 | 0,000486377 |
| ENSMUSG00000004098 | Col5a3 | 0,840596567 | 1,790790496 | 0,010355298 |
| ENSMUSG00000020241 | Col6a2 | 0,533106529 | 1,447041732 | 0,048060386 |
| ENSMUSG00000068196 | Col8a1 | 1,377011659 | 2,597298194 | 0,003767148 |
| ENSMUSG00000091803 | Cox16 | 0,846741591 | 1,798434468 | 0,035970981 |
| ENSMUSG00000039070 | Cpa4 | -1,79765713 | 0,287641326 | 0,017162841 |
| ENSMUSG00000020183 | Cpm | 0,892640124 | 1,856570531 | 0,000245938 |
| ENSMUSG00000040860 | Crocc | 0,633828551 | 1,551677297 | 0,006624821 |
| ENSMUSG00000003345 | Csnk1g2 | 0,405804554 | 1,324827526 | 0,038319992 |
| ENSMUSG00000032515 | Csrnp1 | 0,506000452 | 1,420107803 | 0,013397487 |
| ENSMUSG00000063063 | Ctnna2 | -0,514990842 | 0,699797375 | 0,019482484 |
| ENSMUSG00000044365 | Cxxc4 | -0,5561206 | 0,68012857 | 6,65908E-05 |
| ENSMUSG00000046668 | Cxxc5 | 0,493985009 | 1,408329593 | 0,000230913 |
| ENSMUSG00000024087 | Cyp1b1 | 0,889512716 | 1,852550301 | 0,012183762 |
| ENSMUSG00000024987 | Cyp26a1 | 0,546226957 | 1,460261713 | 0,018133554 |
| ENSMUSG00000062432 | Cyp26c1 | 1,055220349 | 2,07803557 | 0,000117944 |
| ENSMUSG00000086296 | D030055H07Rik | -0,761169431 | 0,590017876 | 0,005795804 |
| ENSMUSG00000092627 | D130058E05Rik | 0,97207819 | 1,961664322 | 0,008916768 |
| ENSMUSG00000097003 | D930007P13Rik | 7,730893277 | 212,4372984 | 2,29878E-10 |
| ENSMUSG00000031285 | Dcx | -0,602393924 | 0,658660106 | 0,023225236 |
| ENSMUSG00000059213 | Ddn | 1,031185072 | 2,043702319 | 0,000334705 |
| ENSMUSG00000041544 | Disp3 | 1,929494151 | 3,809216141 | 5,88737E-15 |
| ENSMUSG00000024868 | Dkk1 | 0,590706036 | 1,505983575 | 0,046254671 |
| ENSMUSG00000036766 | Dner | -1,020607751 | 0,492908665 | 0,042387429 |
| ENSMUSG00000035000 | Dpp4 | 1,671765343 | 3,186042128 | 0,033805298 |
| ENSMUSG00000050272 | Dscam | -1,314703544 | 0,402008094 | 0,00260243 |
| ENSMUSG00000026544 | Dusp23 | 0,746550854 | 1,677776861 | 0,007362108 |
| ENSMUSG00000031530 | Dusp4 | 0,592373693 | 1,507725396 | 7,39026E-06 |
| ENSMUSG00000020888 | Dvl2 | 0,409511245 | 1,328235759 | 0,011318552 |
| ENSMUSG00000068617 | Efcab1 | -0,530360766 | 0,692381573 | 0,03975499 |
| ENSMUSG00000063600 | Egfem1 | -1,572647494 | 0,336190883 | 0,000334705 |
| ENSMUSG00000042302 | Ehbp1 | -0,447231241 | 0,733449102 | 0,000659444 |
| ENSMUSG00000034164 | Emid1 | 0,824797936 | 1,771286934 | 2,08025E-05 |
| ENSMUSG00000029163 | Emilin1 | 0,486454462 | 1,400997578 | 0,001014497 |
| ENSMUSG00000030208 | Emp1 | 0,591977964 | 1,507311887 | 0,001655485 |
| ENSMUSG00000058665 | En1 | 0,807245235 | 1,749866954 | 0,002938985 |
| ENSMUSG00000006445 | Epha2 | 0,477795908 | 1,392614459 | 0,01612813 |
| ENSMUSG00000052504 | Epha3 | -0,91878621 | 0,52895386 | 3,06766E-05 |
| ENSMUSG00000029245 | Epha5 | -0,827500148 | 0,563504819 | 0,000252812 |
| ENSMUSG00000032537 | Ephb1 | -0,563812979 | 0,676511808 | 0,000146246 |
| ENSMUSG00000018166 | Erbb3 | 0,582275493 | 1,497208862 | 0,021635943 |
| ENSMUSG00000062209 | Erbb4 | -0,733598441 | 0,601401995 | 0,002755509 |
| ENSMUSG00000040640 | Erc2 | -0,516873743 | 0,698884646 | 0,045880739 |
| ENSMUSG00000040857 | Erf | 0,401672547 | 1,321038531 | 0,030050973 |
| ENSMUSG00000028967 | Errfi1 | 0,423785879 | 1,341443117 | 0,006921326 |
| ENSMUSG00000024955 | Esrra | 0,478635474 | 1,393425117 | 0,033340939 |
| ENSMUSG00000050212 | Eva1b | 1,061432094 | 2,087002161 | 2,39174E-05 |
| ENSMUSG00000010461 | Eya4 | -0,44417839 | 0,735002779 | 0,006624821 |
| ENSMUSG00000038235 | F11r | 0,710059049 | 1,635871072 | 0,003015724 |
| ENSMUSG00000021750 | Fam107a | -0,901764407 | 0,535231746 | 0,031377407 |
| ENSMUSG00000032657 | Fam189b | 0,462123671 | 1,377568129 | 0,003129822 |
| ENSMUSG00000022358 | Fbxo32 | 1,311031578 | 2,481188904 | 8,81434E-07 |
| ENSMUSG00000003420 | Fcgrt | 0,651339078 | 1,570625339 | 0,006911324 |
| ENSMUSG00000097336 | Fendrr | -0,859937294 | 0,550976505 | 0,018133554 |
| ENSMUSG00000021743 | Fezf2 | -1,373666532 | 0,385909233 | 1,01344E-07 |
| ENSMUSG00000031073 | Fgf15 | -0,766209078 | 0,58796041 | 0,000716342 |
| ENSMUSG00000047632 | Fgfbp3 | 0,958427703 | 1,943190986 | 7,8923E-13 |
| ENSMUSG00000041842 | Fhdc1 | 1,155993596 | 2,22837741 | 7,45977E-09 |
| ENSMUSG00000068699 | Flnc | 0,66256101 | 1,582890009 | 2,25032E-06 |
| ENSMUSG00000001334 | Fndc5 | 0,879418477 | 1,839633631 | 0,04782778 |
| ENSMUSG00000059246 | Foxb1 | 0,686436138 | 1,609303173 | 0,00260243 |
| ENSMUSG00000067261 | Foxd3 | 2,076470532 | 4,217741075 | 8,18946E-10 |
| ENSMUSG00000020950 | Foxg1 | -1,287573593 | 0,409639405 | 0,037881502 |
| ENSMUSG00000029581 | Fscn1 | 0,415372878 | 1,333643325 | 0,036305773 |
| ENSMUSG00000055373 | Fut9 | -0,773101881 | 0,585157996 | 0,001072881 |
| ENSMUSG00000081683 | Fzd10 | 0,902250436 | 1,8689791 | 7,14383E-12 |
| ENSMUSG00000045005 | Fzd5 | -1,105387405 | 0,464777648 | 2,16082E-09 |
| ENSMUSG00000072844 | G530011O06Rik | -1,183926164 | 0,440152033 | 0,03975499 |
| ENSMUSG00000005232 | G6pc2 | 8,897947502 | 477,0337408 | 0,034429012 |
| ENSMUSG00000026787 | Gad2 | -1,902941189 | 0,267397672 | 1,3076E-08 |
| ENSMUSG00000033751 | Gadd45gip1 | 1,06627994 | 2,094026849 | 0,004455553 |
| ENSMUSG00000020766 | Galk1 | 0,489763734 | 1,404214893 | 5,04212E-05 |
| ENSMUSG00000067724 | Gbx1 | 1,203651606 | 2,303219016 | 1,2922E-05 |
| ENSMUSG00000034486 | Gbx2 | 0,887719166 | 1,850248652 | 0,023401282 |
| ENSMUSG00000021943 | Gdf10 | 0,900635979 | 1,866888777 | 0,018133554 |
| ENSMUSG00000019359 | Gdpd2 | -0,592348017 | 0,663262554 | 0,049146822 |
| ENSMUSG00000024366 | Gfra3 | 2,135881373 | 4,395055467 | 5,62731E-06 |
| ENSMUSG00000006345 | Ggt1 | 0,949149068 | 1,930733535 | 0,008602345 |
| ENSMUSG00000046352 | Gjb2 | 1,462572267 | 2,755993083 | 0,000133194 |
| ENSMUSG00000040055 | Gjb6 | 2,956892699 | 7,764498249 | 4,18102E-08 |
| ENSMUSG00000020258 | Glyctk | -0,893413767 | 0,538338769 | 0,018133554 |
| ENSMUSG00000099907 | Gm10421 | -0,720457219 | 0,606905072 | 0,035031556 |
| ENSMUSG00000075589 | Gm11536 | 5,720847845 | 52,74081081 | 0,000157056 |
| ENSMUSG00000086043 | Gm12473 | 1,933200451 | 3,819014641 | 0,018575722 |
| ENSMUSG00000086096 | Gm12688 | 1,927884776 | 3,804969201 | 0,00026592 |
| ENSMUSG00000084897 | Gm14226 | 4,322205517 | 20,00384625 | 0,002287415 |
| ENSMUSG00000086166 | Gm14342 | -0,63670613 | 0,643179741 | 0,021009625 |
| ENSMUSG00000085015 | Gm14424 | 4,860851069 | 29,05774955 | 0,00283498 |
| ENSMUSG00000087626 | Gm15050 | 5,001020604 | 32,02264572 | 1,95637E-13 |
| ENSMUSG00000097621 | Gm26562 | 1,995078008 | 3,986376594 | 0,049146822 |
| ENSMUSG00000097253 | Gm26770 | -1,002194845 | 0,499239903 | 0,016323311 |
| ENSMUSG00000097248 | Gm2694 | -0,827736121 | 0,563412657 | 0,013133577 |
| ENSMUSG00000098172 | Gm26973 | -1,909443922 | 0,266195129 | 0,008602345 |
| ENSMUSG00000100642 | Gm28230 | 10,447864 | 1396,755706 | 0,005473264 |
| ENSMUSG00000101588 | Gm28265 | 3,32263861 | 10,00492613 | 3,28719E-05 |
| ENSMUSG00000101356 | Gm28876 | 2,735699606 | 6,660819124 | 0,004208069 |
| ENSMUSG00000108986 | Gm32061 | -0,526726817 | 0,694127784 | 0,025449507 |
| ENSMUSG00000108616 | Gm35040 | -1,453358497 | 0,365170342 | 0,030323215 |
| ENSMUSG00000067017 | Gm3608 | -3,578712695 | 0,083695119 | 1,63794E-31 |
| ENSMUSG00000104046 | Gm37567 | 3,447136824 | 10,90665519 | 3,67323E-07 |
| ENSMUSG00000103115 | Gm37584 | -0,830819099 | 0,562209954 | 0,049223871 |
| ENSMUSG00000103831 | Gm37608 | -2,922573918 | 0,131891736 | 0,002841013 |
| ENSMUSG00000097156 | Gm3764 | -1,423949898 | 0,372690539 | 0,002054055 |
| ENSMUSG00000109936 | Gm45889 | 2,296481111 | 4,912580712 | 7,97891E-07 |
| ENSMUSG00000078706 | Gm53 | 4,135788321 | 17,579088 | 1,03563E-05 |
| ENSMUSG00000000544 | Gpa33 | 2,096694166 | 4,277281522 | 0,000883896 |
| ENSMUSG00000050668 | Gpatch11 | -0,411499421 | 0,751841564 | 0,045606469 |
| ENSMUSG00000031119 | Gpc4 | -0,462419807 | 0,725767919 | 0,007201195 |
| ENSMUSG00000031517 | Gpm6a | -0,96850478 | 0,51103543 | 0,000132824 |
| ENSMUSG00000018339 | Gpx3 | 0,765156816 | 1,699554724 | 1,53577E-10 |
| ENSMUSG00000042942 | Greb1l | 0,600985145 | 1,516751927 | 0,020195894 |
| ENSMUSG00000074934 | Grem1 | 0,718590531 | 1,645573574 | 0,007389516 |
| ENSMUSG00000050105 | Grrp1 | 1,042984337 | 2,060485537 | 1,8578E-06 |
| ENSMUSG00000022575 | Gsdmd | 1,252195739 | 2,382036862 | 0,034429012 |
| ENSMUSG00000042638 | Gucy2c | 1,95995123 | 3,890488271 | 0,04255026 |
| ENSMUSG00000044927 | H1fx | 0,779387896 | 1,716402487 | 0,01870428 |
| ENSMUSG00000073411 | H2-D1/H2-D1 | 0,823273211 | 1,769415923 | 0,006158577 |
| ENSMUSG00000060586 | H2-Eb1 | 1,522875015 | 2,873631391 | 0,045782643 |
| ENSMUSG00000079507 | H2-Q1 | 2,250522039 | 4,758550029 | 0,019999933 |
| ENSMUSG00000054128 | H2-T3/H2-T3 | 2,948313834 | 7,718464317 | 0,010266237 |
| ENSMUSG00000075277 | Haglr | 6,595363316 | 96,69459318 | 1,61517E-17 |
| ENSMUSG00000075254 | Heg1 | -0,478168395 | 0,717888457 | 0,000781218 |
| ENSMUSG00000047171 | Helt | -4,576074528 | 0,041924154 | 3,93057E-08 |
| ENSMUSG00000028946 | Hes3 | 0,772053059 | 1,707698232 | 0,000101874 |
| ENSMUSG00000087658 | Hotairm1 | 2,923804218 | 7,588444653 | 1,49266E-05 |
| ENSMUSG00000029844 | Hoxa1 | 3,101124227 | 8,58087178 | 4,78532E-11 |
| ENSMUSG00000000938 | Hoxa10 | 9,466809717 | 707,6095542 | 0,025449507 |
| ENSMUSG00000014704 | Hoxa2 | 1,196587141 | 2,29196838 | 6,44298E-06 |
| ENSMUSG00000079560 | Hoxa3 | 6,565707557 | 94,72724731 | 2,36462E-28 |
| ENSMUSG00000000942 | Hoxa4 | 10,76650999 | 1741,977161 | 0,003671368 |
| ENSMUSG00000038253 | Hoxa5 | 12,48496045 | 5732,546548 | 0,000298235 |
| ENSMUSG00000043219 | Hoxa6 | 9,734175529 | 851,6846445 | 0,016192151 |
| ENSMUSG00000038236 | Hoxa7 | 12,06717949 | 4291,241822 | 0,001166958 |
| ENSMUSG00000038227 | Hoxa9 | 8,269469062 | 308,5732224 | 1,7982E-05 |
| ENSMUSG00000056445 | Hoxaas2 | 1,66053589 | 3,161339312 | 3,22008E-06 |
| ENSMUSG00000085696 | Hoxaas3 | 8,786325361 | 441,5170562 | 9,84607E-14 |
| ENSMUSG00000075588 | Hoxb2 | 2,465843774 | 5,524499524 | 6,37424E-13 |
| ENSMUSG00000048763 | Hoxb3 | 4,981542329 | 31,59320355 | 1,06423E-30 |
| ENSMUSG00000084844 | Hoxb3os | 1,862947274 | 3,637500064 | 0,000334705 |
| ENSMUSG00000038692 | Hoxb4 | 7,420700244 | 171,3378694 | 1,12162E-31 |
| ENSMUSG00000038700 | Hoxb5 | 11,38820411 | 2680,347078 | 0,001600756 |
| ENSMUSG00000085645 | Hoxb5os | 13,85033625 | 14769,52951 | 4,18687E-05 |
| ENSMUSG00000000690 | Hoxb6 | 12,59238691 | 6175,699813 | 0,000252812 |
| ENSMUSG00000038721 | Hoxb7 | 9,871290656 | 936,6009271 | 8,02708E-09 |
| ENSMUSG00000056648 | Hoxb8 | 10,44699156 | 1395,911302 | 5,86412E-10 |
| ENSMUSG00000020875 | Hoxb9 | 8,596680564 | 387,1316806 | 6,92063E-10 |
| ENSMUSG00000022484 | Hoxc10 | 9,564554811 | 757,2127067 | 0,018928671 |
| ENSMUSG00000075394 | Hoxc4 | 9,138754449 | 563,6885487 | 4,74393E-13 |
| ENSMUSG00000022485 | Hoxc5 | 11,87944848 | 3767,648032 | 0,000768748 |
| ENSMUSG00000001661 | Hoxc6 | 9,872972936 | 937,6937041 | 2,25999E-14 |
| ENSMUSG00000001657 | Hoxc8 | 10,95786032 | 1989,045165 | 6,59772E-11 |
| ENSMUSG00000036139 | Hoxc9 | 12,60866558 | 6245,778029 | 0,000252812 |
| ENSMUSG00000042448 | Hoxd1 | 4,172550203 | 18,03278354 | 3,09637E-05 |
| ENSMUSG00000079277 | Hoxd3 | 9,529908633 | 739,2449296 | 2,4149E-08 |
| ENSMUSG00000052371 | Hoxd3os1 | 9,659640146 | 808,8005374 | 0,014961624 |
| ENSMUSG00000101174 | Hoxd4 | 9,561734555 | 755,7339135 | 2,4149E-08 |
| ENSMUSG00000027102 | Hoxd8 | 5,677855052 | 51,19230467 | 1,43054E-07 |
| ENSMUSG00000043342 | Hoxd9 | 11,09493637 | 2187,301486 | 0,002738252 |
| ENSMUSG00000025396 | Hsd17b6 | 3,397648544 | 10,53887192 | 0,012428186 |
| ENSMUSG00000025757 | Hspa4l | -0,532271417 | 0,691465215 | 1,96659E-05 |
| ENSMUSG00000028763 | Hspg2 | 0,540634547 | 1,454612165 | 0,037424602 |
| ENSMUSG00000042745 | Id1 | 0,677773235 | 1,599668799 | 1,84246E-05 |
| ENSMUSG00000007872 | Id3 | 0,520324272 | 1,434277592 | 5,0012E-05 |
| ENSMUSG00000021379 | Id4 | -1,585449363 | 0,333220863 | 0,004221621 |
| ENSMUSG00000053560 | Ier2 | 0,718398499 | 1,645354551 | 0,000687309 |
| ENSMUSG00000089762 | Ier5l | 0,971818415 | 1,961311132 | 0,010011259 |
| ENSMUSG00000025491 | Ifitm1 | 1,957376789 | 3,883552015 | 2,91267E-06 |
| ENSMUSG00000038034 | Igsf8 | 0,625842995 | 1,543112233 | 0,000746196 |
| ENSMUSG00000040612 | Ildr2 | -0,455389891 | 0,729313047 | 5,07131E-06 |
| ENSMUSG00000048782 | Insc | 0,668894418 | 1,589854146 | 0,034398853 |
| ENSMUSG00000044030 | Irf2bp1 | 0,515493863 | 1,429483397 | 0,013744264 |
| ENSMUSG00000001504 | Irx2 | -0,491398465 | 0,711335236 | 0,033087944 |
| ENSMUSG00000031734 | Irx3 | 0,446526424 | 1,362755199 | 0,045880739 |
| ENSMUSG00000074766 | Ism1 | 1,217549276 | 2,325513438 | 7,6973E-06 |
| ENSMUSG00000019139 | Isyna1 | 0,406220408 | 1,325209461 | 0,026190329 |
| ENSMUSG00000025321 | Itgb8 | -0,478649732 | 0,717648982 | 0,019412925 |
| ENSMUSG00000002799 | Jag2 | 0,559701663 | 1,473964383 | 0,01029103 |
| ENSMUSG00000052837 | Junb | 1,380225355 | 2,603090293 | 0,021631405 |
| ENSMUSG00000055675 | Kbtbd11 | 0,412239503 | 1,330749942 | 0,001845241 |
| ENSMUSG00000040896 | Kcnd3 | -0,71558923 | 0,608956369 | 0,021044845 |
| ENSMUSG00000033854 | Kcnk10 | -0,802243765 | 0,57345661 | 0,010189211 |
| ENSMUSG00000016346 | Kcnq2 | -0,67511371 | 0,626282855 | 0,005356101 |
| ENSMUSG00000022629 | Kif21a | -0,693536374 | 0,618336308 | 1,26902E-05 |
| ENSMUSG00000024301 | Kifc5b | 0,42253075 | 1,340276583 | 0,008916768 |
| ENSMUSG00000019966 | Kitl | 0,41501087 | 1,333308723 | 0,017879594 |
| ENSMUSG00000020911 | Krt19 | 1,136256281 | 2,198098871 | 3,58219E-07 |
| ENSMUSG00000049382 | Krt8 | 0,558904751 | 1,473150423 | 0,002981196 |
| ENSMUSG00000015647 | Lama5 | 0,718748507 | 1,645753775 | 0,005269947 |
| ENSMUSG00000079330 | Lemd1 | -0,604250758 | 0,657812917 | 0,008053344 |
| ENSMUSG00000001123 | Lgals9 | 1,075219617 | 2,107042796 | 0,03975499 |
| ENSMUSG00000054263 | Lifr | 0,443380163 | 1,35978651 | 0,012960537 |
| ENSMUSG00000050966 | Lin28a | 0,577635994 | 1,492401788 | 0,018434529 |
| ENSMUSG00000049556 | Lingo1 | 0,895096993 | 1,859734912 | 0,000848697 |
| ENSMUSG00000047786 | Lix1 | -0,648504258 | 0,637941369 | 0,003129822 |
| ENSMUSG00000020782 | Llgl2 | 0,736929446 | 1,666624907 | 0,007586918 |
| ENSMUSG00000036111 | Lmo1 | -0,598385889 | 0,660492512 | 0,019482484 |
| ENSMUSG00000030600 | Lrfn1 | 0,595424136 | 1,510916713 | 0,034551371 |
| ENSMUSG00000034648 | Lrrn1 | -0,639604692 | 0,641888807 | 4,08035E-06 |
| ENSMUSG00000001247 | Lsr | 0,588979954 | 1,504182851 | 0,031377407 |
| ENSMUSG00000035342 | Lzts2 | 0,647797979 | 1,566774963 | 0,005375853 |
| ENSMUSG00000057777 | Mab21l2 | -0,651606076 | 0,636571258 | 0,01201795 |
| ENSMUSG00000074622 | Mafb | 0,761645839 | 1,695423676 | 0,027474686 |
| ENSMUSG00000059401 | Mamld1 | -0,712933861 | 0,610078223 | 0,042750452 |
| ENSMUSG00000052727 | Map1b | -0,41475851 | 0,750145049 | 0,013642278 |
| ENSMUSG00000019261 | Map1s | 0,687449131 | 1,610433548 | 0,006567069 |
| ENSMUSG00000015222 | Map2 | -0,569232864 | 0,673975071 | 0,013302911 |
| ENSMUSG00000004054 | Map3k11 | 0,542986253 | 1,45698523 | 0,010014612 |
| ENSMUSG00000018411 | Mapt | -0,740164608 | 0,598671042 | 0,010014612 |
| ENSMUSG00000025732 | Mcrip2 | 0,677572637 | 1,599446389 | 0,018386209 |
| ENSMUSG00000021596 | Mctp1 | -1,364537113 | 0,388359022 | 0,000506171 |
| ENSMUSG00000002968 | Med25 | 0,408707653 | 1,327496128 | 0,031082887 |
| ENSMUSG00000001493 | Meox1 | 1,805943048 | 3,496576447 | 1,6132E-05 |
| ENSMUSG00000009376 | Met | 0,966738857 | 1,954417731 | 0,000196145 |
| ENSMUSG00000048696 | Mex3d | 0,4261262 | 1,343620954 | 0,004183009 |
| ENSMUSG00000025227 | Mfsd13a | 0,524084461 | 1,438020718 | 0,029004304 |
| ENSMUSG00000033307 | Mif | 0,560231136 | 1,47450543 | 0,000569915 |
| ENSMUSG00000065519 | Mir10a | 8,797863426 | 445,0622804 | 0,03975499 |
| ENSMUSG00000097023 | Mir9-3hg | -1,38641507 | 0,382514125 | 7,08039E-06 |
| ENSMUSG00000028496 | Mllt3 | 0,402006202 | 1,321344086 | 0,006790613 |
| ENSMUSG00000001566 | Mnx1 | 9,864929022 | 932,4800319 | 0,012183762 |
| ENSMUSG00000052396 | Mogat2 | 0,560307612 | 1,474583595 | 0,004257507 |
| ENSMUSG00000020000 | Moxd1 | 0,646722452 | 1,56560737 | 0,001166958 |
| ENSMUSG00000041708 | Mpped1 | -0,798241351 | 0,575049738 | 0,000807957 |
| ENSMUSG00000048450 | Msx1 | 0,737373104 | 1,667137507 | 5,86412E-10 |
| ENSMUSG00000019982 | Myb | -0,556908914 | 0,679757037 | 0,041178985 |
| ENSMUSG00000030739 | Myh14 | 0,605401171 | 1,521401751 | 0,025550878 |
| ENSMUSG00000035441 | Myo1d | 0,895737884 | 1,860561249 | 0,000586716 |
| ENSMUSG00000024388 | Myo7b | 1,23757153 | 2,358012767 | 0,040473052 |
| ENSMUSG00000001053 | N4bp3 | 0,56755009 | 1,482004769 | 0,005356101 |
| ENSMUSG00000029413 | Naaa | 0,746454954 | 1,677665338 | 0,019211303 |
| ENSMUSG00000002341 | Ncan | -0,753305146 | 0,593242908 | 0,025008679 |
| ENSMUSG00000047586 | Nccrp1 | 1,524400782 | 2,876672096 | 2,68418E-05 |
| ENSMUSG00000049690 | Nckap5 | -0,55453051 | 0,680878598 | 0,020817396 |
| ENSMUSG00000023009 | Nckap5l | 0,69010841 | 1,613404752 | 0,01084302 |
| ENSMUSG00000071014 | Ndufb6 | 0,608599113 | 1,524777898 | 0,004444599 |
| ENSMUSG00000020153 | Ndufs7 | 0,505702377 | 1,419814426 | 0,010242244 |
| ENSMUSG00000021365 | Nedd9 | 0,747573326 | 1,678966362 | 0,000101874 |
| ENSMUSG00000004891 | Nes | -0,408911616 | 0,753191375 | 0,03975499 |
| ENSMUSG00000030595 | Nfkbib | 0,650710761 | 1,569941456 | 0,018403125 |
| ENSMUSG00000000120 | Ngfr | 0,719116654 | 1,646173792 | 1,19692E-07 |
| ENSMUSG00000021806 | Nid2 | 0,572886193 | 1,487496417 | 0,000581782 |
| ENSMUSG00000021068 | Nin | 0,46471049 | 1,380040389 | 0,024214227 |
| ENSMUSG00000031661 | Nkd1 | 0,445798018 | 1,362067327 | 0,00260243 |
| ENSMUSG00000001496 | Nkx2-1 | -1,647400328 | 0,31921485 | 1,94283E-16 |
| ENSMUSG00000038745 | Nlrp6 | 3,615293516 | 12,25495699 | 0,023325172 |
| ENSMUSG00000042988 | Notum | 0,793013264 | 1,732689643 | 0,015737924 |
| ENSMUSG00000026241 | Nppc | 2,101499304 | 4,29155148 | 0,034148935 |
| ENSMUSG00000022206 | Npr3 | 0,525986199 | 1,439917544 | 0,001492283 |
| ENSMUSG00000060601 | Nr1h2 | 0,431116608 | 1,348276704 | 0,020195894 |
| ENSMUSG00000019803 | Nr2e1 | -0,976463776 | 0,508223934 | 4,18687E-05 |
| ENSMUSG00000025810 | Nrp1 | 0,932220518 | 1,908210752 | 9,82227E-05 |
| ENSMUSG00000025969 | Nrp2 | 0,480903218 | 1,395617137 | 0,003472987 |
| ENSMUSG00000046178 | Nxph1 | 1,97125751 | 3,921097484 | 0,002701217 |
| ENSMUSG00000022026 | Olfm4 | 2,278261576 | 4,850930723 | 0,001544166 |
| ENSMUSG00000027848 | Olfml3 | 0,702829649 | 1,627694163 | 0,000106436 |
| ENSMUSG00000027584 | Oprl1 | -0,838203004 | 0,559339839 | 0,00303584 |
| ENSMUSG00000031173 | Otc | 2,987011823 | 7,928301479 | 0,010588954 |
| ENSMUSG00000005917 | Otx1 | -0,748943003 | 0,595039356 | 0,014350143 |
| ENSMUSG00000022186 | Oxct1 | -0,456833946 | 0,728583412 | 0,021560412 |
| ENSMUSG00000051048 | P4ha3 | 1,338199416 | 2,528355654 | 0,001362981 |
| ENSMUSG00000027508 | Pag1 | 0,676139532 | 1,597858364 | 0,000716342 |
| ENSMUSG00000031284 | Pak3 | -0,972230598 | 0,509717363 | 3,5606E-09 |
| ENSMUSG00000030602 | Pak4 | 0,425108911 | 1,34267386 | 0,027914991 |
| ENSMUSG00000028370 | Pappa | -0,584976121 | 0,666660373 | 0,00216078 |
| ENSMUSG00000073530 | Pappa2 | -0,848075976 | 0,555525108 | 0,005976475 |
| ENSMUSG00000026976 | Pax8 | 0,785121401 | 1,723237321 | 0,01201795 |
| ENSMUSG00000051323 | Pcdh19 | -0,594064143 | 0,662474054 | 0,000506171 |
| ENSMUSG00000021587 | Pcsk1 | 1,252597583 | 2,382700439 | 0,001406602 |
| ENSMUSG00000020388 | Pdlim4 | 0,490528924 | 1,404959871 | 0,001645494 |
| ENSMUSG00000021493 | Pdlim7 | 0,453449275 | 1,369310168 | 0,001241149 |
| ENSMUSG00000024227 | Pdzph1 | -1,05367928 | 0,481738028 | 0,002811007 |
| ENSMUSG00000036218 | Pdzrn4 | -0,773967939 | 0,584806828 | 0,034148935 |
| ENSMUSG00000054728 | Phactr1 | 0,449438143 | 1,365508357 | 0,040644583 |
| ENSMUSG00000010607 | Pigyl | 0,716726412 | 1,643448686 | 0,038498585 |
| ENSMUSG00000024247 | Pkdcc | 0,437222491 | 1,353995074 | 2,08025E-05 |
| ENSMUSG00000023913 | Pla2g7 | 1,092144754 | 2,131907369 | 3,06766E-05 |
| ENSMUSG00000034330 | Plcg2 | 0,451435722 | 1,367400371 | 0,049844099 |
| ENSMUSG00000022565 | Plec | 0,532174827 | 1,446107525 | 0,034429012 |
| ENSMUSG00000030867 | Plk1 | 0,422163235 | 1,339935202 | 0,036436407 |
| ENSMUSG00000031146 | Plp2 | 0,447178106 | 1,36337091 | 0,013293502 |
| ENSMUSG00000074785 | Plxnc1 | -0,596444948 | 0,661381709 | 0,000476574 |
| ENSMUSG00000024521 | Pmaip1 | 1,510089045 | 2,848276184 | 2,72447E-11 |
| ENSMUSG00000031727 | Pmfbp1 | -1,070539735 | 0,476140834 | 0,035970981 |
| ENSMUSG00000042179 | Pnliprp1 | 8,787919751 | 442,0052671 | 0,03975499 |
| ENSMUSG00000027750 | Postn | 0,517183601 | 1,431158641 | 0,007896278 |
| ENSMUSG00000039457 | Ppl | 0,79876233 | 1,739608099 | 0,025550878 |
| ENSMUSG00000037166 | Ppp1r14a | 0,734157173 | 1,663425407 | 0,002124167 |
| ENSMUSG00000029725 | Ppp1r35 | 0,68008429 | 1,602233363 | 0,018679437 |
| ENSMUSG00000050271 | Prag1 | 0,559765177 | 1,474029274 | 0,020648634 |
| ENSMUSG00000079466 | Prdm12 | 0,778721529 | 1,715609879 | 1,23654E-05 |
| ENSMUSG00000040478 | Prdm13 | 1,659339784 | 3,158719402 | 0,005269947 |
| ENSMUSG00000069378 | Prdm6 | 1,249883107 | 2,37822153 | 0,025449507 |
| ENSMUSG00000035456 | Prdm8 | 1,995165045 | 3,986617096 | 0,000581782 |
| ENSMUSG00000036158 | Prickle1 | 0,547525996 | 1,461577162 | 0,02590384 |
| ENSMUSG00000030350 | Prmt8 | -0,548593955 | 0,683686122 | 0,03745545 |
| ENSMUSG00000036106 | Prr5 | 0,442813483 | 1,359252501 | 0,031275409 |
| ENSMUSG00000036030 | Prtg | 0,569930115 | 1,484451661 | 0,044769845 |
| ENSMUSG00000024347 | Psd2 | 0,629901212 | 1,547459028 | 1,42577E-07 |
| ENSMUSG00000005625 | Psmd4 | -0,598601768 | 0,660393686 | 6,52159E-08 |
| ENSMUSG00000023972 | Ptk7 | 0,429774206 | 1,34702274 | 0,011279598 |
| ENSMUSG00000035429 | Ptprh | 2,913085327 | 7,532273191 | 0,013993591 |
| ENSMUSG00000068748 | Ptprz1 | -1,116253764 | 0,4612901 | 5,86412E-10 |
| ENSMUSG00000029576 | Radil | 0,661856204 | 1,582116902 | 0,045782643 |
| ENSMUSG00000037992 | Rara | 0,711273358 | 1,637248556 | 1,84246E-05 |
| ENSMUSG00000020374 | Rasgef1c | 2,398311443 | 5,271857752 | 0,006624821 |
| ENSMUSG00000027510 | Rbm38 | 0,531463712 | 1,445394903 | 0,0344236 |
| ENSMUSG00000070780 | Rbm47 | 0,848481065 | 1,800604169 | 0,035557905 |
| ENSMUSG00000040134 | Rdh7 | 3,27802828 | 9,7002927 | 0,011024962 |
| ENSMUSG00000030110 | Ret | 0,93138621 | 1,907107556 | 0,044769845 |
| ENSMUSG00000020282 | Rhbdf1 | 0,434969739 | 1,351882476 | 0,01201795 |
| ENSMUSG00000054364 | Rhob | 0,466415314 | 1,381672138 | 2,78977E-06 |
| ENSMUSG00000024925 | Rnaseh2c | 0,587438321 | 1,502576371 | 0,033833126 |
| ENSMUSG00000066800 | Rnasel | -0,641792623 | 0,640916082 | 0,026190329 |
| ENSMUSG00000054855 | Rnd1 | 0,511910934 | 1,425937683 | 0,04253553 |
| ENSMUSG00000035890 | Rnf126 | 0,58655389 | 1,501655513 | 0,002966466 |
| ENSMUSG00000031438 | Rnf128 | 1,117807186 | 2,170168685 | 0,001387815 |
| ENSMUSG00000047747 | Rnf150 | -0,444690429 | 0,734741959 | 0,00154561 |
| ENSMUSG00000044164 | Rnf182 | -0,596719547 | 0,661255835 | 0,039702454 |
| ENSMUSG00000035305 | Ror1 | 0,400403678 | 1,319877172 | 0,01201795 |
| ENSMUSG00000021464 | Ror2 | 0,629922211 | 1,547481553 | 2,26929E-05 |
| ENSMUSG00000032238 | Rora | -0,589222431 | 0,664701064 | 0,035970981 |
| ENSMUSG00000046364 | Rpl27a | 0,567269136 | 1,481716188 | 0,006830093 |
| ENSMUSG00000007892 | Rplp1 | 0,614280783 | 1,53079467 | 0,00216078 |
| ENSMUSG00000025508 | Rplp2 | 0,638328596 | 1,556524835 | 0,002637064 |
| ENSMUSG00000092837 | Rpph1 | 1,764131041 | 3,396693495 | 0,035197142 |
| ENSMUSG00000090862 | Rps13 | 0,411175768 | 1,329769108 | 0,031377407 |
| ENSMUSG00000008668 | Rps18 | -0,571952389 | 0,672705804 | 0,001415 |
| ENSMUSG00000061024 | Rrs1 | 0,404908975 | 1,324005371 | 0,000405904 |
| ENSMUSG00000034009 | Rxfp1 | 2,842140446 | 7,170831642 | 0,001660336 |
| ENSMUSG00000031665 | Sall1 | 0,52143492 | 1,435382185 | 0,004144219 |
| ENSMUSG00000002565 | Scin | 2,236317175 | 4,711926956 | 0,040910042 |
| ENSMUSG00000019124 | Scrn1 | -0,585948323 | 0,666211276 | 0,000111986 |
| ENSMUSG00000038580 | Sct | 1,583853238 | 2,997694241 | 0,000306163 |
| ENSMUSG00000007279 | Scube2 | 1,106147796 | 2,152700771 | 9,36286E-05 |
| ENSMUSG00000017009 | Sdc4 | 0,468539102 | 1,383707592 | 0,005375853 |
| ENSMUSG00000053317 | Sec61b | 0,506024409 | 1,420131385 | 0,045306239 |
| ENSMUSG00000076437 | Selenoh | 0,493837674 | 1,408185775 | 0,047837927 |
| ENSMUSG00000075702 | Selenom | 0,644519834 | 1,56321892 | 0,020195894 |
| ENSMUSG00000064373 | Selenop | 0,518082847 | 1,432050975 | 0,005941372 |
| ENSMUSG00000057969 | Sema3b | 0,686829352 | 1,609741857 | 0,02216969 |
| ENSMUSG00000001227 | Sema6b | 0,477751757 | 1,392571842 | 0,034148935 |
| ENSMUSG00000078348 | Sf3b5 | 0,741729508 | 1,672179252 | 0,000186874 |
| ENSMUSG00000061186 | Sfmbt2 | 0,663783718 | 1,584232103 | 0,035970981 |
| ENSMUSG00000027996 | Sfrp2 | 0,509390718 | 1,423448915 | 0,012341468 |
| ENSMUSG00000040666 | Sh3bgr | 1,791596206 | 3,461977158 | 5,17932E-05 |
| ENSMUSG00000033256 | Shf | 0,419615245 | 1,337570788 | 0,006158577 |
| ENSMUSG00000002633 | Shh | -0,430468435 | 0,742020816 | 0,026245261 |
| ENSMUSG00000044461 | Shisa2 | -0,548975326 | 0,683505416 | 0,004731426 |
| ENSMUSG00000050010 | Shisa3 | -1,235027293 | 0,424834462 | 4,40353E-05 |
| ENSMUSG00000041362 | Shtn1 | -1,154874603 | 0,44910522 | 8,78845E-08 |
| ENSMUSG00000024134 | Six2 | -0,739827981 | 0,598810747 | 0,001913767 |
| ENSMUSG00000038805 | Six3 | -1,188852206 | 0,438651709 | 4,32141E-09 |
| ENSMUSG00000093460 | Six3os1 | -1,692083985 | 0,309479556 | 3,26167E-14 |
| ENSMUSG00000021099 | Six6 | -1,129110124 | 0,457197645 | 0,000186874 |
| ENSMUSG00000059182 | Skap2 | 1,166747409 | 2,24504974 | 6,37974E-10 |
| ENSMUSG00000029050 | Ski | 0,412872165 | 1,331333641 | 0,002400049 |
| ENSMUSG00000029700 | Slc13a1 | 3,450199028 | 10,92982978 | 0,016748154 |
| ENSMUSG00000025557 | Slc15a1 | 2,177018495 | 4,522180242 | 0,034324351 |
| ENSMUSG00000032902 | Slc16a1 | 0,428472879 | 1,345808258 | 0,007863826 |
| ENSMUSG00000025161 | Slc16a3 | 0,497396456 | 1,411663719 | 0,016169869 |
| ENSMUSG00000030500 | Slc17a6 | -1,166530401 | 0,445491435 | 0,013993591 |
| ENSMUSG00000005089 | Slc1a2 | -0,500816863 | 0,706706526 | 0,0499222 |
| ENSMUSG00000027219 | Slc28a2 | 2,53784351 | 5,807203176 | 0,026967799 |
| ENSMUSG00000028645 | Slc2a1 | 0,611386608 | 1,527726837 | 5,20778E-09 |
| ENSMUSG00000066152 | Slc31a2 | 0,531285621 | 1,44521649 | 0,007526097 |
| ENSMUSG00000020838 | Slc6a4 | 1,743185005 | 3,347734241 | 0,004845909 |
| ENSMUSG00000036123 | Slc9a3 | 2,51544935 | 5,717757167 | 0,045613411 |
| ENSMUSG00000020733 | Slc9a3r1 | 0,832258954 | 1,78047102 | 9,21631E-10 |
| ENSMUSG00000025020 | Slit1 | 0,582070162 | 1,496995788 | 0,003497452 |
| ENSMUSG00000036790 | Slitrk2 | 0,87783532 | 1,837615996 | 0,001127049 |
| ENSMUSG00000036867 | Smad6 | 0,7662621 | 1,700857293 | 0,001587941 |
| ENSMUSG00000045667 | Smtnl2 | 0,442975164 | 1,35940484 | 0,025800417 |
| ENSMUSG00000042821 | Snai1 | 0,52929969 | 1,443228456 | 0,007502028 |
| ENSMUSG00000034891 | Sncb | 1,131690739 | 2,191153779 | 0,01226937 |
| ENSMUSG00000023045 | Soat2 | 2,024383488 | 4,068179926 | 0,006880141 |
| ENSMUSG00000031626 | Sorbs2 | -0,498596324 | 0,707795098 | 0,01689638 |
| ENSMUSG00000001494 | Sost | 0,983564975 | 1,977345501 | 0,024568287 |
| ENSMUSG00000033006 | Sox10 | 1,228794754 | 2,343711114 | 3,65503E-12 |
| ENSMUSG00000046470 | Sox18 | 0,590005198 | 1,50525217 | 0,013790516 |
| ENSMUSG00000041540 | Sox5 | -0,418578111 | 0,748161633 | 0,014868248 |
| ENSMUSG00000075304 | Sp5 | 0,877387339 | 1,837045474 | 0,002701217 |
| ENSMUSG00000026207 | Speg | 0,418525168 | 1,336560523 | 0,003604504 |
| ENSMUSG00000027315 | Spint1 | 0,603092116 | 1,518968672 | 0,019482484 |
| ENSMUSG00000010154 | Spire2 | 0,493532924 | 1,407888346 | 0,036866497 |
| ENSMUSG00000056222 | Spock1 | -0,946077715 | 0,519041675 | 0,012960537 |
| ENSMUSG00000030257 | Srgap3 | -0,506204142 | 0,70407248 | 0,003740401 |
| ENSMUSG00000070003 | Ssbp4 | 0,59617652 | 1,51170488 | 0,026043977 |
| ENSMUSG00000079478 | Sssca1 | 0,474445347 | 1,389383959 | 0,0344236 |
| ENSMUSG00000028327 | Stra6l | 2,819346833 | 7,058427606 | 0,037254898 |
| ENSMUSG00000039615 | Stub1 | 0,485482579 | 1,400054103 | 0,001284327 |
| ENSMUSG00000053025 | Sv2b | 1,186892522 | 2,276618449 | 9,64116E-05 |
| ENSMUSG00000035864 | Syt1 | -0,921057879 | 0,528121625 | 0,021635943 |
| ENSMUSG00000062327 | T | 1,590384987 | 3,011296961 | 0,014841756 |
| ENSMUSG00000026547 | Tagln2 | 0,412298607 | 1,330804461 | 0,024322051 |
| ENSMUSG00000028417 | Tal2 | -0,978835742 | 0,507389039 | 9,0666E-06 |
| ENSMUSG00000009097 | Tbx1 | 1,000791139 | 2,001097052 | 0,00209663 |
| ENSMUSG00000031965 | Tbx20 | -0,8942485 | 0,538027381 | 0,002252535 |
| ENSMUSG00000087516 | Tbx3os1 | -1,689497217 | 0,310034954 | 4,08035E-06 |
| ENSMUSG00000018263 | Tbx5 | -3,876687878 | 0,06807704 | 0,015076778 |
| ENSMUSG00000000782 | Tcf7 | 0,477198575 | 1,392037981 | 2,8892E-05 |
| ENSMUSG00000024985 | Tcf7l2 | -0,625428326 | 0,648227295 | 0,001636439 |
| ENSMUSG00000034917 | Tjp3 | 0,67666297 | 1,598438204 | 0,031083312 |
| ENSMUSG00000025572 | Tmc6 | 0,577291752 | 1,492045728 | 0,03975499 |
| ENSMUSG00000024736 | Tmem132a | 0,481300933 | 1,396001927 | 0,031082887 |
| ENSMUSG00000034324 | Tmem132c | 0,568339661 | 1,482816076 | 7,85745E-10 |
| ENSMUSG00000020701 | Tmem132e | 0,615694583 | 1,53229554 | 0,002287415 |
| ENSMUSG00000057716 | Tmem178b | -0,579977112 | 0,66897439 | 0,011309443 |
| ENSMUSG00000022857 | Tmprss15 | 3,825345511 | 14,17567483 | 0,019560845 |
| ENSMUSG00000000385 | Tmprss2 | 1,820118934 | 3,531103073 | 0,000442217 |
| ENSMUSG00000036019 | Tmtc2 | -0,56632152 | 0,67533652 | 0,000230913 |
| ENSMUSG00000023915 | Tnfrsf21 | 0,410749261 | 1,329376044 | 0,029089942 |
| ENSMUSG00000039477 | Tnrc18 | 0,584017752 | 1,499018047 | 0,0358502 |
| ENSMUSG00000032741 | Tpcn1 | 0,419855311 | 1,33779338 | 0,020464204 |
| ENSMUSG00000020308 | Tpgs1 | 1,115095929 | 2,166094116 | 6,80515E-05 |
| ENSMUSG00000002043 | Trappc6a | 0,602623628 | 1,518475497 | 0,000365309 |
| ENSMUSG00000038812 | Trmt112 | 0,408628829 | 1,3274236 | 0,023112081 |
| ENSMUSG00000029723 | Tsc22d4 | 0,523864833 | 1,437801818 | 0,009716022 |
| ENSMUSG00000046982 | Tshz1 | 1,077680444 | 2,110639877 | 2,57736E-11 |
| ENSMUSG00000021217 | Tshz3 | 1,27122984 | 2,413672338 | 5,78232E-10 |
| ENSMUSG00000042345 | Ubash3a | 1,615572961 | 3,064332741 | 0,013744264 |
| ENSMUSG00000025876 | Unc5a | 1,010832483 | 2,015073529 | 0,000508125 |
| ENSMUSG00000006313 | Upk1a | 1,376697438 | 2,596732561 | 0,000110503 |
| ENSMUSG00000019820 | Utrn | 0,457517332 | 1,373176746 | 0,02216969 |
| ENSMUSG00000006270 | Vax1 | -2,591842688 | 0,165873729 | 6,06681E-06 |
| ENSMUSG00000085794 | Vax2os | -2,693793209 | 0,15455656 | 0,031443567 |
| ENSMUSG00000049641 | Vgll2 | 1,177931597 | 2,262521649 | 0,021950461 |
| ENSMUSG00000024076 | Vit | -0,698760141 | 0,61610146 | 0,003278249 |
| ENSMUSG00000021239 | Vsx2 | -2,691789102 | 0,15477141 | 5,86412E-10 |
| ENSMUSG00000040389 | Wdr47 | -0,418363439 | 0,748272967 | 0,02216969 |
| ENSMUSG00000023336 | Wfdc1 | -0,942201411 | 0,520438137 | 0,001598364 |
| ENSMUSG00000071192 | Wfikkn1 | 1,045286419 | 2,06377604 | 3,20586E-17 |
| ENSMUSG00000022997 | Wnt1 | 1,498921593 | 2,82631368 | 5,48096E-21 |
| ENSMUSG00000027840 | Wnt2b | 0,818811375 | 1,763952088 | 0,002434329 |
| ENSMUSG00000036856 | Wnt4 | 0,605679686 | 1,521695489 | 0,002287415 |
| ENSMUSG00000033227 | Wnt6 | 0,708202486 | 1,633767269 | 0,001362981 |
| ENSMUSG00000012282 | Wnt8a | 2,979893797 | 7,889280848 | 0,015745777 |
| ENSMUSG00000016458 | Wt1 | 2,749034811 | 6,722672226 | 0,020817396 |
| ENSMUSG00000049090 | Zadh2 | 0,785020412 | 1,723116699 | 5,6814E-11 |
| ENSMUSG00000022708 | Zbtb20 | -0,566470365 | 0,675266848 | 0,032333146 |
| ENSMUSG00000021127 | Zfp36l1 | 0,603669105 | 1,519576289 | 0,034551371 |
| ENSMUSG00000039081 | Zfp503 | 0,954648265 | 1,938107057 | 4,22028E-15 |
| ENSMUSG00000085795 | Zfp703 | 0,464539567 | 1,379876898 | 0,000581782 |
| ENSMUSG00000054716 | Zfp771 | 0,6321967 | 1,549923167 | 0,013351283 |
| ENSMUSG00000053390 | Zfp952 | -0,460087396 | 0,726942221 | 0,007032758 |
