# Supplementary material for: Severe head dysgenesis resulting from imbalance between anterior and posterior ontogenetic programs
Source: Cell Death Dis. 2019 Oct 24;10(11):812. doi: 10.1038/s41419-019-2040-0 (PMC6813351; doi:10.1038/s41419-019-2040-0)
Supplement: Supplementary file 2 — Table S2 [file 41419_2019_2040_MOESM2_ESM.docx]

**Table S2 sheet 1**

Principal component analysis of the differentially-expressed genes in the head of *CDX2*-expressing *vs* control E10.5 littermates.

| **ensGeneID** | **Gene Symbol** | **PC1** | **PC2** | **Category** |
| --- | --- | --- | --- | --- |
|  |  |  |  |  |
| ENSMUSG00000043773 | 1700048O20Rik | 0,174034 | -0,47014 | 1 |
| ENSMUSG00000074415 | 2610203C20Rik | 3,8387 | -0,52291 | 1 |
| ENSMUSG00000021098 | 4930447C04Rik | -0,67961 | -0,61868 | 1 |
| ENSMUSG00000102995 | A330074H02Rik | -2,36623 | -0,77605 | 1 |
| ENSMUSG00000095041 | AC149090.1 | 3,226255 | -0,44752 | 1 |
| ENSMUSG00000025964 | Adam23 | -0,20185 | -0,50184 | 1 |
| ENSMUSG00000109311 | AI314278 | -2,96108 | -0,57151 | 1 |
| ENSMUSG00000053279 | Aldh1a1 | -0,98219 | -1,11594 | 1 |
| ENSMUSG00000024747 | Aldh1a7 | -1,41082 | -0,76325 | 1 |
| ENSMUSG00000032826 | Ank2 | 2,822828 | -0,39543 | 1 |
| ENSMUSG00000031075 | Ano1 | 1,276072 | -0,83294 | 1 |
| ENSMUSG00000020052 | Ascl1 | 1,048402 | -0,75921 | 1 |
| ENSMUSG00000097428 | AW047730 | -2,4414 | -0,73623 | 1 |
| ENSMUSG00000000861 | Bcl11a | -0,44657 | -0,76554 | 1 |
| ENSMUSG00000004110 | Cacna1e | -1,10143 | -0,59093 | 1 |
| ENSMUSG00000050840 | Cdh20 | -1,14242 | -0,58026 | 1 |
| ENSMUSG00000026312 | Cdh7 | -1,40336 | -0,60624 | 1 |
| ENSMUSG00000036510 | Cdh8 | -1,71103 | -0,8024 | 1 |
| ENSMUSG00000002107 | Celf2 | 3,48923 | -0,47375 | 1 |
| ENSMUSG00000044288 | Cnr1 | -0,42613 | -0,55161 | 1 |
| ENSMUSG00000030092 | Cntn6 | -1,75916 | -0,65449 | 1 |
| ENSMUSG00000039070 | Cpa4 | -2,71795 | -0,71889 | 1 |
| ENSMUSG00000063063 | Ctnna2 | 1,187013 | -0,54382 | 1 |
| ENSMUSG00000044365 | Cxxc4 | 2,28716 | -0,55346 | 1 |
| ENSMUSG00000086296 | D030055H07Rik | -1,09935 | -0,63732 | 1 |
| ENSMUSG00000031285 | Dcx | 2,905412 | -0,61257 | 1 |
| ENSMUSG00000036766 | Dner | -1,52522 | -0,72131 | 1 |
| ENSMUSG00000050272 | Dscam | -2,35286 | -0,65373 | 1 |
| ENSMUSG00000068617 | Efcab1 | -0,80811 | -0,51568 | 1 |
| ENSMUSG00000063600 | Egfem1 | -2,38736 | -0,68925 | 1 |
| ENSMUSG00000042302 | Ehbp1 | 2,453057 | -0,47895 | 1 |
| ENSMUSG00000052504 | Epha3 | 1,097938 | -0,82095 | 1 |
| ENSMUSG00000029245 | Epha5 | -0,74566 | -0,65333 | 1 |
| ENSMUSG00000032537 | Ephb1 | 0,749239 | -0,55309 | 1 |
| ENSMUSG00000062209 | Erbb4 | -0,60749 | -0,6461 | 1 |
| ENSMUSG00000040640 | Erc2 | -0,74335 | -0,47224 | 1 |
| ENSMUSG00000010461 | Eya4 | 0,535317 | -0,47516 | 1 |
| ENSMUSG00000021750 | Fam107a | -2,18703 | -0,58303 | 1 |
| ENSMUSG00000097336 | Fendrr | -2,01283 | -0,55055 | 1 |
| ENSMUSG00000021743 | Fezf2 | -0,83044 | -0,94538 | 1 |
| ENSMUSG00000031073 | Fgf15 | 1,45302 | -0,75282 | 1 |
| ENSMUSG00000020950 | Foxg1 | 0,312689 | -1,07973 | 1 |
| ENSMUSG00000055373 | Fut9 | -0,33691 | -0,65125 | 1 |
| ENSMUSG00000045005 | Fzd5 | -0,1362 | -0,80393 | 1 |
| ENSMUSG00000072844 | G530011O06Rik | -1,88782 | -0,73563 | 1 |
| ENSMUSG00000026787 | Gad2 | -2,15288 | -0,83206 | 1 |
| ENSMUSG00000019359 | Gdpd2 | -1,20042 | -0,54823 | 1 |
| ENSMUSG00000020258 | Glyctk | -1,63066 | -0,65877 | 1 |
| ENSMUSG00000099907 | Gm10421 | -1,32225 | -0,61644 | 1 |
| ENSMUSG00000086166 | Gm14342 | -0,95397 | -0,56877 | 1 |
| ENSMUSG00000114133 | Gm20075 | -2,49484 | -0,65084 | 1 |
| ENSMUSG00000097253 | Gm26770 | -1,59202 | -0,69343 | 1 |
| ENSMUSG00000097248 | Gm2694 | -1,65453 | -0,60968 | 1 |
| ENSMUSG00000098172 | Gm26973 | -2,90236 | -0,65989 | 1 |
| ENSMUSG00000108986 | Gm32061 | 0,058219 | -0,53479 | 1 |
| ENSMUSG00000108616 | Gm35040 | -2,36572 | -0,75312 | 1 |
| ENSMUSG00000067017 | Gm3608 | -0,83707 | -1,7252 | 1 |
| ENSMUSG00000103115 | Gm37584 | -1,94131 | -0,61211 | 1 |
| ENSMUSG00000103831 | Gm37608 | -3,12164 | -0,68541 | 1 |
| ENSMUSG00000097156 | Gm3764 | 1,222332 | -1,18296 | 1 |
| ENSMUSG00000050668 | Gpatch11 | 0,821641 | -0,4791 | 1 |
| ENSMUSG00000031119 | Gpc4 | 1,691431 | -0,4979 | 1 |
| ENSMUSG00000031517 | Gpm6a | 0,783524 | -0,85861 | 1 |
| ENSMUSG00000075254 | Heg1 | 3,537474 | -0,46322 | 1 |
| ENSMUSG00000047171 | Helt | -2,99427 | -0,94449 | 1 |
| ENSMUSG00000025757 | Hspa4l | 3,15991 | -0,52559 | 1 |
| ENSMUSG00000021379 | Id4 | 2,194193 | -1,38905 | 1 |
| ENSMUSG00000040612 | Ildr2 | 4,924965 | -0,41268 | 1 |
| ENSMUSG00000001504 | Irx2 | 1,450346 | -0,56437 | 1 |
| ENSMUSG00000025321 | Itgb8 | 2,434347 | -0,49983 | 1 |
| ENSMUSG00000040896 | Kcnd3 | -1,00686 | -0,61416 | 1 |
| ENSMUSG00000033854 | Kcnk10 | -0,93644 | -0,65985 | 1 |
| ENSMUSG00000016346 | Kcnq2 | -0,45988 | -0,58583 | 1 |
| ENSMUSG00000022629 | Kif21a | 3,989852 | -0,63775 | 1 |
| ENSMUSG00000079330 | Lemd1 | 1,002233 | -0,60645 | 1 |
| ENSMUSG00000047786 | Lix1 | 1,958389 | -0,68188 | 1 |
| ENSMUSG00000036111 | Lmo1 | -0,65227 | -0,57876 | 1 |
| ENSMUSG00000034648 | Lrrn1 | 3,912709 | -0,5972 | 1 |
| ENSMUSG00000057777 | Mab21l2 | 0,138793 | -0,64846 | 1 |
| ENSMUSG00000059401 | Mamld1 | -1,17591 | -0,59976 | 1 |
| ENSMUSG00000052727 | Map1b | 7,296962 | -0,29296 | 1 |
| ENSMUSG00000015222 | Map2 | 3,965669 | -0,56973 | 1 |
| ENSMUSG00000018411 | Mapt | 0,321126 | -0,67023 | 1 |
| ENSMUSG00000021596 | Mctp1 | -2,27994 | -0,70494 | 1 |
| ENSMUSG00000097023 | Mir9-3hg | -0,1576 | -1,07247 | 1 |
| ENSMUSG00000041708 | Mpped1 | 0,894034 | -0,75324 | 1 |
| ENSMUSG00000019982 | Myb | -0,68508 | -0,55403 | 1 |
| ENSMUSG00000002341 | Ncan | 0,186975 | -0,72324 | 1 |
| ENSMUSG00000049690 | Nckap5 | -0,05453 | -0,53696 | 1 |
| ENSMUSG00000004891 | Nes | 8,033332 | -0,31495 | 1 |
| ENSMUSG00000001496 | Nkx2-1 | -0,21102 | -1,11992 | 1 |
| ENSMUSG00000019803 | Nr2e1 | 1,706725 | -0,90492 | 1 |
| ENSMUSG00000027584 | Oprl1 | -1,32251 | -0,6409 | 1 |
| ENSMUSG00000005917 | Otx1 | 1,736813 | -0,77643 | 1 |
| ENSMUSG00000022186 | Oxct1 | 3,85128 | -0,47147 | 1 |
| ENSMUSG00000031284 | Pak3 | 0,836064 | -0,83502 | 1 |
| ENSMUSG00000028370 | Pappa | 0,495616 | -0,55644 | 1 |
| ENSMUSG00000073530 | Pappa2 | -1,19391 | -0,65857 | 1 |
| ENSMUSG00000051323 | Pcdh19 | 1,59027 | -0,58347 | 1 |
| ENSMUSG00000024227 | Pdzph1 | -1,37264 | -0,7585 | 1 |
| ENSMUSG00000036218 | Pdzrn4 | -1,8871 | -0,55903 | 1 |
| ENSMUSG00000074785 | Plxnc1 | 0,502669 | -0,53755 | 1 |
| ENSMUSG00000031727 | Pmfbp1 | -2,24372 | -0,63678 | 1 |
| ENSMUSG00000030350 | Prmt8 | 0,239998 | -0,54085 | 1 |
| ENSMUSG00000005625 | Psmd4 | 5,620588 | -0,47139 | 1 |
| ENSMUSG00000068748 | Ptprz1 | 1,797048 | -0,93279 | 1 |
| ENSMUSG00000066800 | Rnasel | -0,0198 | -0,57183 | 1 |
| ENSMUSG00000047747 | Rnf150 | 1,142543 | -0,47602 | 1 |
| ENSMUSG00000044164 | Rnf182 | -1,06151 | -0,55661 | 1 |
| ENSMUSG00000032238 | Rora | -1,22123 | -0,52933 | 1 |
| ENSMUSG00000008668 | Rps18 | 7,417073 | -0,45888 | 1 |
| ENSMUSG00000019124 | Scrn1 | 2,181569 | -0,59291 | 1 |
| ENSMUSG00000112343 | Sfta3-ps | -0,89254 | -1,06781 | 1 |
| ENSMUSG00000002633 | Shh | 0,513757 | -0,48915 | 1 |
| ENSMUSG00000044461 | Shisa2 | 1,94215 | -0,59565 | 1 |
| ENSMUSG00000050010 | Shisa3 | -0,72372 | -0,88173 | 1 |
| ENSMUSG00000041362 | Shtn1 | -1,07997 | -0,79358 | 1 |
| ENSMUSG00000024134 | Six2 | 0,345368 | -0,71148 | 1 |
| ENSMUSG00000038805 | Six3 | 0,07407 | -0,93908 | 1 |
| ENSMUSG00000093460 | Six3os1 | 2,00302 | -1,38707 | 1 |
| ENSMUSG00000021099 | Six6 | -1,21414 | -0,8079 | 1 |
| ENSMUSG00000030500 | Slc17a6 | -1,54557 | -0,7696 | 1 |
| ENSMUSG00000005089 | Slc1a2 | 1,290517 | -0,52002 | 1 |
| ENSMUSG00000031626 | Sorbs2 | 1,398518 | -0,54807 | 1 |
| ENSMUSG00000041540 | Sox5 | 1,106481 | -0,44335 | 1 |
| ENSMUSG00000056222 | Spock1 | -1,44385 | -0,67781 | 1 |
| ENSMUSG00000030257 | Srgap3 | 4,744492 | -0,45001 | 1 |
| ENSMUSG00000035864 | Syt1 | -1,46732 | -0,6813 | 1 |
| ENSMUSG00000028417 | Tal2 | 0,941709 | -0,88271 | 1 |
| ENSMUSG00000031965 | Tbx20 | -1,30171 | -0,64337 | 1 |
| ENSMUSG00000087516 | Tbx3os1 | -2,34464 | -0,71931 | 1 |
| ENSMUSG00000018263 | Tbx5 | -3,37596 | -0,6531 | 1 |
| ENSMUSG00000024985 | Tcf7l2 | 2,128486 | -0,62913 | 1 |
| ENSMUSG00000057716 | Tmem178b | -0,6752 | -0,52616 | 1 |
| ENSMUSG00000036019 | Tmtc2 | 0,864643 | -0,55179 | 1 |
| ENSMUSG00000006270 | Vax1 | -2,53538 | -0,96278 | 1 |
| ENSMUSG00000085794 | Vax2os | -3,29759 | -0,69802 | 1 |
| ENSMUSG00000024076 | Vit | -0,96439 | -0,59708 | 1 |
| ENSMUSG00000021239 | Vsx2 | -2,61491 | -0,86662 | 1 |
| ENSMUSG00000040389 | Wdr47 | 0,77044 | -0,44955 | 1 |
| ENSMUSG00000023336 | Wfdc1 | -0,80998 | -0,75143 | 1 |
| ENSMUSG00000022708 | Zbtb20 | -0,52612 | -0,51918 | 1 |
| ENSMUSG00000053390 | Zfp952 | 0,868117 | -0,50264 | 1 |
|  |  |  |  |  |
| ENSMUSG00000032300 | 1700017B05Rik | 1,355544 | 0,11831 | 2 |
| ENSMUSG00000086841 | 2410006H16Rik | 1,520188 | 0,125913 | 2 |
| ENSMUSG00000096001 | 2610528A11Rik | -3,07351 | 0,390082 | 2 |
| ENSMUSG00000041605 | 5730559C18Rik | 1,245119 | 0,055051 | 2 |
| ENSMUSG00000025194 | Abcc2 | -2,79395 | 0,144209 | 2 |
| ENSMUSG00000003346 | Abhd17a | 2,031665 | 0,133966 | 2 |
| ENSMUSG00000007950 | Abhd8 | 1,616929 | 0,070896 | 2 |
| ENSMUSG00000020681 | Ace | -1,67852 | 0,201718 | 2 |
| ENSMUSG00000062825 | Actg1 | 7,162656 | 0,313584 | 2 |
| ENSMUSG00000049538 | Adamts16 | -2,42137 | 0,00147 | 2 |
| ENSMUSG00000066113 | Adamtsl1 | -2,23469 | 0,04018 | 2 |
| ENSMUSG00000037692 | Ahdc1 | 2,465508 | 0,346647 | 2 |
| ENSMUSG00000011096 | Akt1s1 | 1,688617 | 0,134331 | 2 |
| ENSMUSG00000035561 | Aldh1b1 | -0,82795 | 0,276917 | 2 |
| ENSMUSG00000032531 | Amotl2 | 4,565654 | 0,292976 | 2 |
| ENSMUSG00000021314 | Amph | -0,18304 | -0,01755 | 2 |
| ENSMUSG00000039062 | Anpep | 0,365713 | 0,418538 | 2 |
| ENSMUSG00000004931 | Apba3 | 0,929521 | 0,099209 | 2 |
| ENSMUSG00000079042 | Apela | -2,51792 | 0,234283 | 2 |
| ENSMUSG00000037010 | Apln | -0,01255 | 0,065648 | 2 |
| ENSMUSG00000057315 | Arhgap24 | -1,27637 | 0,283651 | 2 |
| ENSMUSG00000035697 | Arhgap45 | 1,490491 | 0,104007 | 2 |
| ENSMUSG00000004661 | Arid3b | 3,243806 | 0,221388 | 2 |
| ENSMUSG00000029622 | Arpc1b | 1,768938 | 0,17634 | 2 |
| ENSMUSG00000076441 | Ass1 | -1,15524 | 0,135135 | 2 |
| ENSMUSG00000037621 | Atoh8 | -0,27395 | -0,0233 | 2 |
| ENSMUSG00000000142 | Axin2 | 3,376685 | 0,174382 | 2 |
| ENSMUSG00000006731 | B4galnt1 | -1,79651 | -0,01965 | 2 |
| ENSMUSG00000030847 | Bag3 | 0,566747 | 0,038501 | 2 |
| ENSMUSG00000002980 | Bcam | 3,010102 | 0,349967 | 2 |
| ENSMUSG00000040093 | Bmf | 3,377346 | 0,205164 | 2 |
| ENSMUSG00000015943 | Bola1 | -0,40301 | 0,128737 | 2 |
| ENSMUSG00000062638 | Btnl1 | -2,91913 | 0,189256 | 2 |
| ENSMUSG00000024340 | Btnl2 | -3,17877 | 0,011706 | 2 |
| ENSMUSG00000073420 | Btnl5-ps | -3,2511 | 0,103551 | 2 |
| ENSMUSG00000058914 | C1qtnf3 | -2,52075 | 0,212838 | 2 |
| ENSMUSG00000034889 | Cactin | 2,864455 | 0,178075 | 2 |
| ENSMUSG00000032936 | Camkv | 1,074136 | 0,082078 | 2 |
| ENSMUSG00000070372 | Capza1 | 2,827864 | 0,292798 | 2 |
| ENSMUSG00000000805 | Car4 | 0,615851 | 0,6569 | 2 |
| ENSMUSG00000033170 | Card10 | 0,257679 | 0,172842 | 2 |
| ENSMUSG00000063605 | Ccdc102a | 0,323046 | 0,049778 | 2 |
| ENSMUSG00000095098 | Ccdc85b | -1,16428 | 0,152931 | 2 |
| ENSMUSG00000049521 | Cdc42ep1 | 1,06431 | 0,125578 | 2 |
| ENSMUSG00000025497 | Cdhr5 | -2,82228 | 0,147045 | 2 |
| ENSMUSG00000026437 | Cdk18 | -1,02261 | -0,00918 | 2 |
| ENSMUSG00000023067 | Cdkn1a | 0,068964 | 0,01146 | 2 |
| ENSMUSG00000006585 | Cdt1 | 3,364064 | 0,243683 | 2 |
| ENSMUSG00000024619 | Cdx1 | -2,69917 | 0,147176 | 2 |
| ENSMUSG00000074272 | Ceacam1 | -2,42109 | 0,002063 | 2 |
| ENSMUSG00000030077 | Chl1 | -1,48631 | 0,663735 | 2 |
| ENSMUSG00000032997 | Chpf | 1,240902 | 0,241759 | 2 |
| ENSMUSG00000068547 | Clca4a | -2,91336 | 0,098676 | 2 |
| ENSMUSG00000070473 | Cldn3 | -2,10283 | -0,0579 | 2 |
| ENSMUSG00000023959 | Clic5 | -2,43708 | 0,018973 | 2 |
| ENSMUSG00000024330 | Col11a2 | -0,05442 | 0,170788 | 2 |
| ENSMUSG00000040690 | Col16a1 | -0,38257 | 0,053171 | 2 |
| ENSMUSG00000001506 | Col1a1 | 2,876535 | 0,240339 | 2 |
| ENSMUSG00000031274 | Col4a5 | 2,783434 | 0,308619 | 2 |
| ENSMUSG00000031273 | Col4a6 | 1,351671 | 0,496384 | 2 |
| ENSMUSG00000026837 | Col5a1 | 3,549886 | 0,35243 | 2 |
| ENSMUSG00000004098 | Col5a3 | -1,66165 | 0,018143 | 2 |
| ENSMUSG00000020241 | Col6a2 | -0,15106 | 0,01095 | 2 |
| ENSMUSG00000068196 | Col8a1 | -2,35067 | 0,04502 | 2 |
| ENSMUSG00000091803 | Cox16 | -1,92362 | -0,06252 | 2 |
| ENSMUSG00000020183 | Cpm | -0,17265 | 0,270511 | 2 |
| ENSMUSG00000040860 | Crocc | 3,117366 | 0,261153 | 2 |
| ENSMUSG00000003345 | Csnk1g2 | 4,991114 | 0,228874 | 2 |
| ENSMUSG00000032515 | Csrnp1 | -0,41616 | 0,01163 | 2 |
| ENSMUSG00000046668 | Cxxc5 | 2,716881 | 0,182533 | 2 |
| ENSMUSG00000024087 | Cyp1b1 | -0,67933 | 0,216857 | 2 |
| ENSMUSG00000024987 | Cyp26a1 | -0,71799 | -0,0089 | 2 |
| ENSMUSG00000062432 | Cyp26c1 | -0,43908 | 0,286952 | 2 |
| ENSMUSG00000092627 | D130058E05Rik | -2,11193 | -0,05034 | 2 |
| ENSMUSG00000059213 | Ddn | -1,73296 | 0,059573 | 2 |
| ENSMUSG00000041544 | Disp3 | -0,53873 | 0,658208 | 2 |
| ENSMUSG00000024868 | Dkk1 | -0,34385 | 0,069937 | 2 |
| ENSMUSG00000035000 | Dpp4 | -2,31032 | 0,161052 | 2 |
| ENSMUSG00000026544 | Dusp23 | -1,35247 | 0,024937 | 2 |
| ENSMUSG00000031530 | Dusp4 | 1,44353 | 0,218272 | 2 |
| ENSMUSG00000020888 | Dvl2 | 3,139776 | 0,158683 | 2 |
| ENSMUSG00000034164 | Emid1 | 0,559119 | 0,232326 | 2 |
| ENSMUSG00000029163 | Emilin1 | 3,222157 | 0,224562 | 2 |
| ENSMUSG00000030208 | Emp1 | 2,219711 | 0,276089 | 2 |
| ENSMUSG00000058665 | En1 | -0,25007 | 0,108044 | 2 |
| ENSMUSG00000006445 | Epha2 | 0,247729 | 0,048366 | 2 |
| ENSMUSG00000018166 | Erbb3 | 1,493013 | 0,242089 | 2 |
| ENSMUSG00000040857 | Erf | 3,318102 | 0,157814 | 2 |
| ENSMUSG00000028967 | Errfi1 | 1,969811 | 0,146002 | 2 |
| ENSMUSG00000024955 | Esrra | 0,168071 | 0,00163 | 2 |
| ENSMUSG00000050212 | Eva1b | -0,9093 | 0,153937 | 2 |
| ENSMUSG00000038235 | F11r | 1,154374 | 0,305089 | 2 |
| ENSMUSG00000032657 | Fam189b | 1,180581 | 0,101863 | 2 |
| ENSMUSG00000022358 | Fbxo32 | -1,09994 | 0,318259 | 2 |
| ENSMUSG00000003420 | Fcgrt | -0,95583 | 0,008122 | 2 |
| ENSMUSG00000047632 | Fgfbp3 | 1,615576 | 0,405645 | 2 |
| ENSMUSG00000041842 | Fhdc1 | -0,07143 | 0,41638 | 2 |
| ENSMUSG00000068699 | Flnc | 1,532996 | 0,227551 | 2 |
| ENSMUSG00000001334 | Fndc5 | -1,80484 | 0,024949 | 2 |
| ENSMUSG00000059246 | Foxb1 | -0,88264 | 0,014096 | 2 |
| ENSMUSG00000067261 | Foxd3 | -2,02248 | 0,320559 | 2 |
| ENSMUSG00000029581 | Fscn1 | 7,054548 | 0,316129 | 2 |
| ENSMUSG00000081683 | Fzd10 | 3,031061 | 0,507532 | 2 |
| ENSMUSG00000005232 | G6pc2 | -3,41677 | 0,086656 | 2 |
| ENSMUSG00000033751 | Gadd45gip1 | -1,40418 | 0,063899 | 2 |
| ENSMUSG00000020766 | Galk1 | 3,140287 | 0,240736 | 2 |
| ENSMUSG00000067724 | Gbx1 | -1,19019 | 0,222525 | 2 |
| ENSMUSG00000034486 | Gbx2 | -1,89614 | -0,00257 | 2 |
| ENSMUSG00000021943 | Gdf10 | -1,39657 | 0,086795 | 2 |
| ENSMUSG00000024366 | Gfra3 | -2,25215 | 0,281843 | 2 |
| ENSMUSG00000006345 | Ggt1 | -1,46337 | 0,08821 | 2 |
| ENSMUSG00000046352 | Gjb2 | -2,27218 | 0,080901 | 2 |
| ENSMUSG00000040055 | Gjb6 | -2,76645 | 0,188326 | 2 |
| ENSMUSG00000075589 | Gm11536 | -3,19175 | 0,2687 | 2 |
| ENSMUSG00000086043 | Gm12473 | -2,97859 | -0,05018 | 2 |
| ENSMUSG00000086096 | Gm12688 | -2,18645 | 0,262974 | 2 |
| ENSMUSG00000084897 | Gm14226 | -2,58084 | 0,573557 | 2 |
| ENSMUSG00000085015 | Gm14424 | -3,32645 | 0,03308 | 2 |
| ENSMUSG00000087626 | Gm15050 | -2,70883 | 0,471908 | 2 |
| ENSMUSG00000097621 | Gm26562 | -3,0891 | -0,07254 | 2 |
| ENSMUSG00000100642 | Gm28230 | -3,0958 | 0,432384 | 2 |
| ENSMUSG00000101588 | Gm28265 | -3,05642 | 0,076252 | 2 |
| ENSMUSG00000101356 | Gm28876 | -3,08052 | 0,029562 | 2 |
| ENSMUSG00000104046 | Gm37567 | -2,77214 | 0,300573 | 2 |
| ENSMUSG00000109936 | Gm45889 | -2,14359 | 0,367515 | 2 |
| ENSMUSG00000078706 | Gm53 | -2,92072 | 0,3164 | 2 |
| ENSMUSG00000000544 | Gpa33 | -2,6537 | 0,097004 | 2 |
| ENSMUSG00000018339 | Gpx3 | 2,136369 | 0,382006 | 2 |
| ENSMUSG00000042942 | Greb1l | 2,684719 | 0,343963 | 2 |
| ENSMUSG00000074934 | Grem1 | -0,90726 | 0,053974 | 2 |
| ENSMUSG00000050105 | Grrp1 | -0,95979 | 0,19337 | 2 |
| ENSMUSG00000022575 | Gsdmd | -2,33638 | -0,01321 | 2 |
| ENSMUSG00000042638 | Gucy2c | -2,80107 | 0,056695 | 2 |
| ENSMUSG00000044927 | H1fx | -0,04154 | 0,124509 | 2 |
| ENSMUSG00000073411 | H2-D1 | 1,53779 | 0,403923 | 2 |
| ENSMUSG00000060586 | H2-Eb1 | -2,37335 | 0,081155 | 2 |
| ENSMUSG00000079507 | H2-Q1 | -3,04262 | -0,03035 | 2 |
| ENSMUSG00000054128 | H2-T3/H2-T3 | -3,13217 | 0,073376 | 2 |
| ENSMUSG00000028946 | Hes3 | -0,72597 | 0,080165 | 2 |
| ENSMUSG00000087658 | Hotairm1 | -2,88862 | 0,135173 | 2 |
| ENSMUSG00000029844 | Hoxa1 | -2,44523 | 0,388275 | 2 |
| ENSMUSG00000000938 | Hoxa10 | -3,38917 | 0,211177 | 2 |
| ENSMUSG00000014704 | Hoxa2 | -0,9809 | 0,28188 | 2 |
| ENSMUSG00000000942 | Hoxa4 | -3,01312 | 0,524664 | 2 |
| ENSMUSG00000043219 | Hoxa6 | -3,30539 | 0,275431 | 2 |
| ENSMUSG00000056445 | Hoxaas2 | -1,18961 | 0,439499 | 2 |
| ENSMUSG00000075588 | Hoxb2 | -1,84703 | 0,492758 | 2 |
| ENSMUSG00000084844 | Hoxb3os | -2,02877 | 0,289642 | 2 |
| ENSMUSG00000038700 | Hoxb5 | -2,81992 | 0,726468 | 2 |
| ENSMUSG00000022484 | Hoxc10 | -3,3277 | 0,240319 | 2 |
| ENSMUSG00000042448 | Hoxd1 | -3,13954 | 0,110664 | 2 |
| ENSMUSG00000052371 | Hoxd3os1 | -3,28259 | 0,24791 | 2 |
| ENSMUSG00000027102 | Hoxd8 | -2,9055 | 0,452617 | 2 |
| ENSMUSG00000043342 | Hoxd9 | -2,95653 | 0,644662 | 2 |
| ENSMUSG00000025396 | Hsd17b6 | -3,31992 | -0,01223 | 2 |
| ENSMUSG00000028763 | Hspg2 | 5,408721 | 0,399442 | 2 |
| ENSMUSG00000042745 | Id1 | 2,385476 | 0,268632 | 2 |
| ENSMUSG00000007872 | Id3 | 5,404173 | 0,310281 | 2 |
| ENSMUSG00000053560 | Ier2 | 1,901835 | 0,281298 | 2 |
| ENSMUSG00000089762 | Ier5l | -0,04507 | 0,153852 | 2 |
| ENSMUSG00000025491 | Ifitm1 | -1,53966 | 0,478416 | 2 |
| ENSMUSG00000038034 | Igsf8 | 1,12293 | 0,149637 | 2 |
| ENSMUSG00000048782 | Insc | -1,62912 | -0,05936 | 2 |
| ENSMUSG00000044030 | Irf2bp1 | 2,080166 | 0,122766 | 2 |
| ENSMUSG00000031734 | Irx3 | 1,391334 | 0,051706 | 2 |
| ENSMUSG00000074766 | Ism1 | -1,73687 | 0,127365 | 2 |
| ENSMUSG00000019139 | Isyna1 | 3,691747 | 0,161679 | 2 |
| ENSMUSG00000002799 | Jag2 | 1,411668 | 0,18388 | 2 |
| ENSMUSG00000052837 | Junb | -2,30534 | -0,01745 | 2 |
| ENSMUSG00000055675 | Kbtbd11 | 3,124861 | 0,192941 | 2 |
| ENSMUSG00000024301 | Kifc5b | 1,300533 | 0,102162 | 2 |
| ENSMUSG00000019966 | Kitl | 2,136892 | 0,154284 | 2 |
| ENSMUSG00000020911 | Krt19 | -0,29139 | 0,371691 | 2 |
| ENSMUSG00000049382 | Krt8 | 2,03761 | 0,235486 | 2 |
| ENSMUSG00000015647 | Lama5 | 3,502392 | 0,462812 | 2 |
| ENSMUSG00000001123 | Lgals9 | -2,09898 | 0,003501 | 2 |
| ENSMUSG00000054263 | Lifr | 1,774642 | 0,157708 | 2 |
| ENSMUSG00000050966 | Lin28a | 5,720451 | 0,452015 | 2 |
| ENSMUSG00000049556 | Lingo1 | -1,30987 | 0,06365 | 2 |
| ENSMUSG00000020782 | Llgl2 | 0,009538 | 0,190609 | 2 |
| ENSMUSG00000030600 | Lrfn1 | 0,313724 | 0,073924 | 2 |
| ENSMUSG00000001247 | Lsr | -0,64143 | 0,03294 | 2 |
| ENSMUSG00000035342 | Lzts2 | 3,13245 | 0,29161 | 2 |
| ENSMUSG00000074622 | Mafb | -0,54039 | 0,140696 | 2 |
| ENSMUSG00000019261 | Map1s | 2,006297 | 0,267636 | 2 |
| ENSMUSG00000004054 | Map3k11 | 2,619585 | 0,2201 | 2 |
| ENSMUSG00000025732 | Mcrip2 | -1,40792 | -0,02334 | 2 |
| ENSMUSG00000002968 | Med25 | 4,831428 | 0,214077 | 2 |
| ENSMUSG00000001493 | Meox1 | -2,27611 | 0,181072 | 2 |
| ENSMUSG00000009376 | Met | -0,52881 | 0,260731 | 2 |
| ENSMUSG00000048696 | Mex3d | 1,965276 | 0,088411 | 2 |
| ENSMUSG00000025227 | Mfsd13a | -0,4836 | 0,00249 | 2 |
| ENSMUSG00000033307 | Mif | 6,249583 | 0,407199 | 2 |
| ENSMUSG00000065519 | Mir10a | -3,43704 | 0,092719 | 2 |
| ENSMUSG00000028496 | Mllt3 | 3,949825 | 0,232901 | 2 |
| ENSMUSG00000001566 | Mnx1 | -3,24514 | 0,297456 | 2 |
| ENSMUSG00000052396 | Mogat2 | 1,72365 | 0,21399 | 2 |
| ENSMUSG00000020000 | Moxd1 | 0,439383 | 0,163103 | 2 |
| ENSMUSG00000048450 | Msx1 | 3,420948 | 0,41506 | 2 |
| ENSMUSG00000030739 | Myh14 | -1,18229 | -0,03495 | 2 |
| ENSMUSG00000035441 | Myo1d | -1,06462 | 0,126402 | 2 |
| ENSMUSG00000024388 | Myo7b | -2,3028 | 0,02266 | 2 |
| ENSMUSG00000001053 | N4bp3 | 0,277828 | 0,052025 | 2 |
| ENSMUSG00000029413 | Naaa | -1,3255 | 0,047293 | 2 |
| ENSMUSG00000047586 | Nccrp1 | -2,29185 | 0,049286 | 2 |
| ENSMUSG00000023009 | Nckap5l | 1,634889 | 0,245912 | 2 |
| ENSMUSG00000071014 | Ndufb6 | 0,788924 | 0,143918 | 2 |
| ENSMUSG00000020153 | Ndufs7 | 2,188306 | 0,169885 | 2 |
| ENSMUSG00000021365 | Nedd9 | 2,233145 | 0,40299 | 2 |
| ENSMUSG00000030595 | Nfkbib | 0,188662 | 0,098652 | 2 |
| ENSMUSG00000000120 | Ngfr | 1,462226 | 0,282185 | 2 |
| ENSMUSG00000021806 | Nid2 | 3,017868 | 0,294582 | 2 |
| ENSMUSG00000021068 | Nin | 2,988553 | 0,250478 | 2 |
| ENSMUSG00000031661 | Nkd1 | 2,510754 | 0,142713 | 2 |
| ENSMUSG00000038745 | Nlrp6 | -2,6706 | 0,411114 | 2 |
| ENSMUSG00000042988 | Notum | -1,85204 | -0,03818 | 2 |
| ENSMUSG00000026241 | Nppc | -2,93552 | -0,03857 | 2 |
| ENSMUSG00000022206 | Npr3 | 1,360311 | 0,176039 | 2 |
| ENSMUSG00000060601 | Nr1h2 | 2,101386 | 0,10897 | 2 |
| ENSMUSG00000025810 | Nrp1 | 0,806156 | 0,412711 | 2 |
| ENSMUSG00000025969 | Nrp2 | 3,76515 | 0,293 | 2 |
| ENSMUSG00000046178 | Nxph1 | -2,87106 | -0,00339 | 2 |
| ENSMUSG00000022026 | Olfm4 | -2,7968 | 0,105229 | 2 |
| ENSMUSG00000027848 | Olfml3 | 0,036229 | 0,15976 | 2 |
| ENSMUSG00000031173 | Otc | -3,21456 | 0,022061 | 2 |
| ENSMUSG00000051048 | P4ha3 | -2,04202 | 0,116533 | 2 |
| ENSMUSG00000027508 | Pag1 | 0,404682 | 0,205325 | 2 |
| ENSMUSG00000030602 | Pak4 | 2,907659 | 0,150215 | 2 |
| ENSMUSG00000026976 | Pax8 | -1,35276 | -0,00549 | 2 |
| ENSMUSG00000021587 | Pcsk1 | -2,07646 | 0,081359 | 2 |
| ENSMUSG00000020388 | Pdlim4 | 0,527751 | 0,064001 | 2 |
| ENSMUSG00000021493 | Pdlim7 | 2,845178 | 0,167902 | 2 |
| ENSMUSG00000054728 | Phactr1 | 0,638883 | 0,091198 | 2 |
| ENSMUSG00000010607 | Pigyl | -0,88144 | 0,008299 | 2 |
| ENSMUSG00000024247 | Pkdcc | 3,410851 | 0,215417 | 2 |
| ENSMUSG00000023913 | Pla2g7 | -1,01044 | 0,218356 | 2 |
| ENSMUSG00000034330 | Plcg2 | 0,479682 | 0,069028 | 2 |
| ENSMUSG00000022565 | Plec | 3,203118 | 0,271795 | 2 |
| ENSMUSG00000030867 | Plk1 | 4,161284 | 0,206334 | 2 |
| ENSMUSG00000031146 | Plp2 | 0,301954 | 0,045064 | 2 |
| ENSMUSG00000024521 | Pmaip1 | -0,43065 | 0,530665 | 2 |
| ENSMUSG00000042179 | Pnliprp1 | -3,43623 | 0,083431 | 2 |
| ENSMUSG00000027750 | Postn | 0,987858 | 0,127287 | 2 |
| ENSMUSG00000039457 | Ppl | -0,96231 | 0,129461 | 2 |
| ENSMUSG00000037166 | Ppp1r14a | -1,23254 | 0,00621 | 2 |
| ENSMUSG00000029725 | Ppp1r35 | -0,32397 | 0,027275 | 2 |
| ENSMUSG00000050271 | Prag1 | 1,045852 | 0,165045 | 2 |
| ENSMUSG00000079466 | Prdm12 | -0,03542 | 0,177194 | 2 |
| ENSMUSG00000040478 | Prdm13 | -2,54016 | 0,026693 | 2 |
| ENSMUSG00000069378 | Prdm6 | -2,50858 | -0,02387 | 2 |
| ENSMUSG00000035456 | Prdm8 | -2,39116 | 0,170129 | 2 |
| ENSMUSG00000036158 | Prickle1 | 0,883072 | 0,166863 | 2 |
| ENSMUSG00000036106 | Prr5 | -0,22925 | -0,03271 | 2 |
| ENSMUSG00000036030 | Prtg | 5,526551 | 0,444641 | 2 |
| ENSMUSG00000024347 | Psd2 | 0,964545 | 0,190435 | 2 |
| ENSMUSG00000023972 | Ptk7 | 4,812881 | 0,273578 | 2 |
| ENSMUSG00000035429 | Ptprh | -3,23125 | -0,03958 | 2 |
| ENSMUSG00000029576 | Radil | -1,42248 | -0,05313 | 2 |
| ENSMUSG00000037992 | Rara | 1,333272 | 0,26642 | 2 |
| ENSMUSG00000020374 | Rasgef1c | -3,07687 | -0,01454 | 2 |
| ENSMUSG00000027510 | Rbm38 | 1,460128 | 0,130546 | 2 |
| ENSMUSG00000070780 | Rbm47 | -2,10619 | -0,06635 | 2 |
| ENSMUSG00000040134 | Rdh7 | -3,2342 | 0,087897 | 2 |
| ENSMUSG00000030110 | Ret | -0,73516 | 0,233097 | 2 |
| ENSMUSG00000058833 | Rex1bd | 0,424536 | 0,094879 | 2 |
| ENSMUSG00000020282 | Rhbdf1 | 1,842706 | 0,124961 | 2 |
| ENSMUSG00000054364 | Rhob | 3,308732 | 0,216208 | 2 |
| ENSMUSG00000024925 | Rnaseh2c | 0,852893 | 0,078858 | 2 |
| ENSMUSG00000054855 | Rnd1 | -0,81407 | -0,0354 | 2 |
| ENSMUSG00000035890 | Rnf126 | 2,266637 | 0,19604 | 2 |
| ENSMUSG00000031438 | Rnf128 | -1,77059 | 0,093617 | 2 |
| ENSMUSG00000035305 | Ror1 | 1,505303 | 0,097753 | 2 |
| ENSMUSG00000021464 | Ror2 | 1,969296 | 0,268293 | 2 |
| ENSMUSG00000046364 | Rpl27a | 5,705835 | 0,341684 | 2 |
| ENSMUSG00000007892 | Rplp1 | 8,654901 | 0,535492 | 2 |
| ENSMUSG00000025508 | Rplp2 | 7,012686 | 0,45463 | 2 |
| ENSMUSG00000092837 | Rpph1 | -2,55107 | -0,05673 | 2 |
| ENSMUSG00000090862 | Rps13 | 6,244207 | 0,277161 | 2 |
| ENSMUSG00000061024 | Rrs1 | 3,010044 | 0,155206 | 2 |
| ENSMUSG00000034009 | Rxfp1 | -3,08718 | -0,00138 | 2 |
| ENSMUSG00000031665 | Sall1 | 3,424948 | 0,307783 | 2 |
| ENSMUSG00000002565 | Scin | -3,07708 | -0,00738 | 2 |
| ENSMUSG00000038580 | Sct | -2,34585 | 0,078292 | 2 |
| ENSMUSG00000007279 | Scube2 | -0,3871 | 0,361565 | 2 |
| ENSMUSG00000017009 | Sdc4 | 0,276939 | 0,052782 | 2 |
| ENSMUSG00000053317 | Sec61b | 2,952955 | 0,154289 | 2 |
| ENSMUSG00000076437 | Selenoh | 4,871185 | 0,231152 | 2 |
| ENSMUSG00000075702 | Selenom | -0,32296 | 0,022128 | 2 |
| ENSMUSG00000064373 | Selenop | 3,387252 | 0,285324 | 2 |
| ENSMUSG00000057969 | Sema3b | -1,56454 | -0,03724 | 2 |
| ENSMUSG00000001227 | Sema6b | -0,7735 | -0,04806 | 2 |
| ENSMUSG00000078348 | Sf3b5 | 2,182996 | 0,265551 | 2 |
| ENSMUSG00000061186 | Sfmbt2 | -1,17108 | 0,027915 | 2 |
| ENSMUSG00000027996 | Sfrp2 | 3,337886 | 0,223457 | 2 |
| ENSMUSG00000040666 | Sh3bgr | -1,65355 | 0,386868 | 2 |
| ENSMUSG00000033256 | Shf | 1,320087 | 0,089416 | 2 |
| ENSMUSG00000059182 | Skap2 | 0,226462 | 0,457691 | 2 |
| ENSMUSG00000029050 | Ski | 4,359262 | 0,216083 | 2 |
| ENSMUSG00000029700 | Slc13a1 | -3,33909 | 0,015469 | 2 |
| ENSMUSG00000025557 | Slc15a1 | -2,77784 | 0,122977 | 2 |
| ENSMUSG00000032902 | Slc16a1 | 4,642451 | 0,277299 | 2 |
| ENSMUSG00000025161 | Slc16a3 | 4,151811 | 0,319936 | 2 |
| ENSMUSG00000027219 | Slc28a2 | -3,01413 | 0,072186 | 2 |
| ENSMUSG00000028645 | Slc2a1 | 6,217362 | 0,467822 | 2 |
| ENSMUSG00000066152 | Slc31a2 | -0,29584 | 0,033628 | 2 |
| ENSMUSG00000020838 | Slc6a4 | -2,65191 | 0,041512 | 2 |
| ENSMUSG00000036123 | Slc9a3 | -2,96885 | 0,115644 | 2 |
| ENSMUSG00000020733 | Slc9a3r1 | 1,514078 | 0,3726 | 2 |
| ENSMUSG00000025020 | Slit1 | 2,331102 | 0,259178 | 2 |
| ENSMUSG00000036790 | Slitrk2 | -0,38217 | 0,239217 | 2 |
| ENSMUSG00000036867 | Smad6 | -0,49899 | 0,106276 | 2 |
| ENSMUSG00000045667 | Smtnl2 | 0,076933 | 0,030988 | 2 |
| ENSMUSG00000042821 | Snai1 | 0,516626 | 0,06404 | 2 |
| ENSMUSG00000034891 | Sncb | -2,28142 | -0,0206 | 2 |
| ENSMUSG00000023045 | Soat2 | -2,89587 | -0,00641 | 2 |
| ENSMUSG00000001494 | Sost | -1,89332 | 0,005781 | 2 |
| ENSMUSG00000033006 | Sox10 | 1,150869 | 0,601566 | 2 |
| ENSMUSG00000046470 | Sox18 | -0,97689 | -0,00687 | 2 |
| ENSMUSG00000075304 | Sp5 | -1,47285 | 0,027536 | 2 |
| ENSMUSG00000026207 | Speg | 1,816212 | 0,077518 | 2 |
| ENSMUSG00000027315 | Spint1 | -0,6533 | 0,051434 | 2 |
| ENSMUSG00000010154 | Spire2 | -0,92393 | -0,05338 | 2 |
| ENSMUSG00000070003 | Ssbp4 | 0,756017 | 0,089734 | 2 |
| ENSMUSG00000079478 | Sssca1 | 0,426769 | 0,006874 | 2 |
| ENSMUSG00000028327 | Stra6l | -2,41382 | 0,391458 | 2 |
| ENSMUSG00000039615 | Stub1 | 1,485251 | 0,104123 | 2 |
| ENSMUSG00000053025 | Sv2b | -1,63399 | 0,150049 | 2 |
| ENSMUSG00000062327 | T | -2,79496 | -0,03015 | 2 |
| ENSMUSG00000026547 | Tagln2 | 2,850918 | 0,192663 | 2 |
| ENSMUSG00000009097 | Tbx1 | -1,77141 | 0,04149 | 2 |
| ENSMUSG00000000782 | Tcf7 | 1,701062 | 0,128776 | 2 |
| ENSMUSG00000034917 | Tjp3 | -1,05629 | 0,007628 | 2 |
| ENSMUSG00000025572 | Tmc6 | 0,180894 | 0,120995 | 2 |
| ENSMUSG00000024736 | Tmem132a | 3,926973 | 0,250389 | 2 |
| ENSMUSG00000034324 | Tmem132c | 4,351604 | 0,352967 | 2 |
| ENSMUSG00000020701 | Tmem132e | -0,01616 | 0,061796 | 2 |
| ENSMUSG00000022857 | Tmprss15 | -3,38557 | -0,0315 | 2 |
| ENSMUSG00000000385 | Tmprss2 | -2,58676 | 0,078635 | 2 |
| ENSMUSG00000023915 | Tnfrsf21 | 1,982958 | 0,13644 | 2 |
| ENSMUSG00000039477 | Tnrc18 | 4,38536 | 0,349784 | 2 |
| ENSMUSG00000032741 | Tpcn1 | 2,081888 | 0,13436 | 2 |
| ENSMUSG00000020308 | Tpgs1 | -0,81237 | 0,192358 | 2 |
| ENSMUSG00000002043 | Trappc6a | 0,059053 | 0,076241 | 2 |
| ENSMUSG00000038812 | Trmt112 | 0,554186 | -0,00421 | 2 |
| ENSMUSG00000029723 | Tsc22d4 | 1,749954 | 0,132788 | 2 |
| ENSMUSG00000046982 | Tshz1 | 2,038612 | 0,594019 | 2 |
| ENSMUSG00000021217 | Tshz3 | -0,55266 | 0,361532 | 2 |
| ENSMUSG00000042345 | Ubash3a | -2,69853 | 0,011305 | 2 |
| ENSMUSG00000025876 | Unc5a | -1,01717 | 0,138148 | 2 |
| ENSMUSG00000006313 | Upk1a | -2,05547 | 0,117545 | 2 |
| ENSMUSG00000019820 | Utrn | 3,916768 | 0,28891 | 2 |
| ENSMUSG00000049641 | Vgll2 | -2,0289 | 0,022222 | 2 |
| ENSMUSG00000071192 | Wfikkn1 | 1,472784 | 0,493279 | 2 |
| ENSMUSG00000022997 | Wnt1 | -0,35709 | 0,489757 | 2 |
| ENSMUSG00000027840 | Wnt2b | -0,83204 | 0,114532 | 2 |
| ENSMUSG00000036856 | Wnt4 | 0,322922 | 0,139254 | 2 |
| ENSMUSG00000033227 | Wnt6 | -0,5458 | 0,09489 | 2 |
| ENSMUSG00000012282 | Wnt8a | -3,25658 | -0,05423 | 2 |
| ENSMUSG00000016458 | Wt1 | -3,21238 | -0,03877 | 2 |
| ENSMUSG00000049090 | Zadh2 | 1,664903 | 0,350381 | 2 |
| ENSMUSG00000021127 | Zfp36l1 | 2,170609 | 0,233509 | 2 |
| ENSMUSG00000039081 | Zfp503 | 2,334415 | 0,481405 | 2 |
| ENSMUSG00000085795 | Zfp703 | 1,362121 | 0,096535 | 2 |
| ENSMUSG00000054716 | Zfp771 | -0,7268 | -0,00361 | 2 |
|  |  |  |  |  |
| ENSMUSG00000056468 | 5730596B20Rik | -2,64512 | 0,78495 | 3 |
| ENSMUSG00000013584 | Aldh1a2 | -1,07337 | 1,200173 | 3 |
| ENSMUSG00000031326 | Cdx4 | -2,60077 | 1,058899 | 3 |
| ENSMUSG00000022371 | Col14a1 | -1,62617 | 0,766672 | 3 |
| ENSMUSG00000097003 | D930007P13Rik | -2,54758 | 0,841245 | 3 |
| ENSMUSG00000075277 | Haglr | -2,43115 | 0,872248 | 3 |
| ENSMUSG00000079560 | Hoxa3 | -2,06631 | 1,109504 | 3 |
| ENSMUSG00000038253 | Hoxa5 | -2,38271 | 1,176706 | 3 |
| ENSMUSG00000038236 | Hoxa7 | -2,61107 | 1,006118 | 3 |
| ENSMUSG00000038227 | Hoxa9 | -2,12755 | 1,429176 | 3 |
| ENSMUSG00000085696 | Hoxaas3 | -2,07239 | 1,345971 | 3 |
| ENSMUSG00000048763 | Hoxb3 | -2,09204 | 0,901723 | 3 |
| ENSMUSG00000038692 | Hoxb4 | -1,74416 | 1,471916 | 3 |
| ENSMUSG00000085645 | Hoxb5os | -1,67768 | 1,914957 | 3 |
| ENSMUSG00000000690 | Hoxb6 | -2,33993 | 1,234647 | 3 |
| ENSMUSG00000038721 | Hoxb7 | -2,0783 | 1,429273 | 3 |
| ENSMUSG00000056648 | Hoxb8 | -1,7606 | 1,747607 | 3 |
| ENSMUSG00000020875 | Hoxb9 | -1,57213 | 1,919192 | 3 |
| ENSMUSG00000075394 | Hoxc4 | -1,61845 | 1,85794 | 3 |
| ENSMUSG00000022485 | Hoxc5 | -2,63697 | 0,907922 | 3 |
| ENSMUSG00000001661 | Hoxc6 | -1,50834 | 1,976045 | 3 |
| ENSMUSG00000001657 | Hoxc8 | -1,47409 | 2,056624 | 3 |
| ENSMUSG00000036139 | Hoxc9 | -2,34084 | 1,249103 | 3 |
| ENSMUSG00000079277 | Hoxd3 | -2,22452 | 1,223791 | 3 |
| ENSMUSG00000101174 | Hoxd4 | -2,21489 | 1,243162 | 3 |

**Table S2 sheet 2**

Common genes between those upregulated in the head of *CDX2*-expressing E10.5 embryos and those downregulated in *Cdx*-null E8 embryos (1^st^ column), between those upregulated in the head of *CDX2*-expressing E10.5 embryos and those upregulated in epiblast stem cells (eSC) cultured with CHIR99021 + Fgf8 (2^nd^ column), and between those upregulated in the head of *CDX2*-expressing E10.5 embryos, those downregulated in *Cdx*-null E8 embryos and those upregulated in epiblast stem cells (eSC) cultured with CHIR99021 + Fgf8 (3^rd^ column).

| ***Cdx2* head up *vs* *Cdx* KO down** | ***Cdx2* head up *vs* eSC Fgf+Ch up** | ***Cdx2* head up *vs* *Cdx* KO down *vs* eSC Fgf+Ch up** |
| --- | --- | --- |
|  |  |  |
| 2410006H16Rik | 2410006H16Rik | 2410006H16Rik |
| 2610528A11Rik | 2610528A11Rik | 2610528A11Rik |
| Arid3b | Aldh1a2 | Arid3b |
| Cdx1 | Arid3b | Dusp4 |
| Cdx4 | Axin2 | Foxb1 |
| Dusp4 | Cdkn1a | Hoxb8 |
| F11r | Cyp1b1 | Hoxb9 |
| Foxb1 | Cyp26a1 | Hoxd9 |
| Gadd45gip1 | Dkk1 | Mllt3 |
| Gfra3 | Dusp4 | T |
| Hoxa5 | Emid1 | Tmem132c |
| Hoxa9 | Foxb1 |  |
| Hoxb7 | Fscn1 |  |
| Hoxb8 | Fzd10 |  |
| Hoxb9 | Greb1l |  |
| Hoxc4 | Hoxa1 |  |
| Hoxc5 | Hoxb2 |  |
| Hoxc6 | Hoxb3 |  |
| Hoxc9 | Hoxb4 |  |
| Hoxd1 | Hoxb8 |  |
| Hoxd4 | Hoxb9 |  |
| Hoxd9 | Hoxd9 |  |
| Ifitm1 | Id1 |  |
| Isyna1 | Id3 |  |
| Map1s | Ifitm1 |  |
| Mllt3 | Ism1 |  |
| Naaa | Kbtbd11 |  |
| Prickle1 | Mllt3 |  |
| Ptk7 | Mogat2 |  |
| Smtnl2 | Nkd1 |  |
| Sp5 | Notum |  |
| Spint1 | Nrp2 |  |
| Sv2b | Pdlim4 |  |
| T | Pla2g7 |  |
| Tmem132c | Prtg |  |
| Upk1a | Rhob |  |
|  | Rpl27a |  |
|  | Rplp1 |  |
|  | Slc16a1 |  |
|  | Snai1 |  |
|  | Sp5 |  |
|  | T |  |
|  | Tmem132c |  |
|  | Wnt6 |  |
|  | Zfp36l1 |  |
|  | Zfp503 |  |
|  | Zfp703 |  |

**Table S2 sheet 3**

Differentially-expressed transcription regulator genes in the head of *CDX2*-expressing *vs* control E10.5 littermates.

| **ensGeneID** | **Gene Symbol** | **log2FoldChange** | **Fold Change** | **adjp** |
| --- | --- | --- | --- | --- |
|  |  |  |  |  |
| ENSMUSG00000037692 | Ahdc1 | 0,692972193 | 1,616610578 | 0,006830093 |
| ENSMUSG00000004661 | Arid3b | 0,422636099 | 1,340374457 | 0,013871909 |
| ENSMUSG00000020052 | Ascl1 | -0,732478709 | 0,601868948 | 0,021187512 |
| ENSMUSG00000037621 | Atoh8 | 0,435214551 | 1,352111898 | 0,025727664 |
| ENSMUSG00000000861 | Bcl11a | -0,867286381 | 0,548176968 | 0,040686599 |
| ENSMUSG00000095098 | Ccdc85b | 1,257781382 | 2,391277204 | 0,000794633 |
| ENSMUSG00000024619 | Cdx1 | 2,717953829 | 6,579389983 | 2,96688E-06 |
| ENSMUSG00000031326 | Cdx4 | 12,21085689 | 4740,609664 | 0,001587941 |
| ENSMUSG00000032515 | Csrnp1 | 0,506000452 | 1,420107803 | 0,013397487 |
| ENSMUSG00000058665 | En1 | 0,807245235 | 1,749866954 | 0,002938985 |
| ENSMUSG00000040857 | Erf | 0,401672547 | 1,321038531 | 0,030050973 |
| ENSMUSG00000024955 | Esrra | 0,478635474 | 1,393425117 | 0,033340939 |
| ENSMUSG00000010461 | Eya4 | -0,44417839 | 0,735002779 | 0,006624821 |
| ENSMUSG00000021743 | Fezf2 | -1,373666532 | 0,385909233 | 1,01344E-07 |
| ENSMUSG00000059246 | Foxb1 | 0,686436138 | 1,609303173 | 0,00260243 |
| ENSMUSG00000067261 | Foxd3 | 2,076470532 | 4,217741075 | 8,18946E-10 |
| ENSMUSG00000020950 | Foxg1 | -1,287573593 | 0,409639405 | 0,037881502 |
| ENSMUSG00000067724 | Gbx1 | 1,203651606 | 2,303219016 | 1,2922E-05 |
| ENSMUSG00000034486 | Gbx2 | 0,887719166 | 1,850248652 | 0,023401282 |
| ENSMUSG00000047171 | Helt | -4,576074528 | 0,041924154 | 3,93057E-08 |
| ENSMUSG00000028946 | Hes3 | 0,772053059 | 1,707698232 | 0,000101874 |
| ENSMUSG00000029844 | Hoxa1 | 3,101124227 | 8,58087178 | 4,78532E-11 |
| ENSMUSG00000000938 | Hoxa10 | 9,466809717 | 707,6095542 | 0,025449507 |
| ENSMUSG00000014704 | Hoxa2 | 1,196587141 | 2,29196838 | 6,44298E-06 |
| ENSMUSG00000079560 | Hoxa3 | 6,565707557 | 94,72724731 | 2,36462E-28 |
| ENSMUSG00000000942 | Hoxa4 | 10,76650999 | 1741,977161 | 0,003671368 |
| ENSMUSG00000038253 | Hoxa5 | 12,48496045 | 5732,546548 | 0,000298235 |
| ENSMUSG00000043219 | Hoxa6 | 9,734175529 | 851,6846445 | 0,016192151 |
| ENSMUSG00000038236 | Hoxa7 | 12,06717949 | 4291,241822 | 0,001166958 |
| ENSMUSG00000038227 | Hoxa9 | 8,269469062 | 308,5732224 | 1,7982E-05 |
| ENSMUSG00000075588 | Hoxb2 | 2,465843774 | 5,524499524 | 6,37424E-13 |
| ENSMUSG00000048763 | Hoxb3 | 4,981542329 | 31,59320355 | 1,06423E-30 |
| ENSMUSG00000038692 | Hoxb4 | 7,420700244 | 171,3378694 | 1,12162E-31 |
| ENSMUSG00000038700 | Hoxb5 | 11,38820411 | 2680,347078 | 0,001600756 |
| ENSMUSG00000000690 | Hoxb6 | 12,59238691 | 6175,699813 | 0,000252812 |
| ENSMUSG00000038721 | Hoxb7 | 9,871290656 | 936,6009271 | 8,02708E-09 |
| ENSMUSG00000056648 | Hoxb8 | 10,44699156 | 1395,911302 | 5,86412E-10 |
| ENSMUSG00000020875 | Hoxb9 | 8,596680564 | 387,1316806 | 6,92063E-10 |
| ENSMUSG00000022484 | Hoxc10 | 9,564554811 | 757,2127067 | 0,018928671 |
| ENSMUSG00000075394 | Hoxc4 | 9,138754449 | 563,6885487 | 4,74393E-13 |
| ENSMUSG00000022485 | Hoxc5 | 11,87944848 | 3767,648032 | 0,000768748 |
| ENSMUSG00000001661 | Hoxc6 | 9,872972936 | 937,6937041 | 2,25999E-14 |
| ENSMUSG00000001657 | Hoxc8 | 10,95786032 | 1989,045165 | 6,59772E-11 |
| ENSMUSG00000036139 | Hoxc9 | 12,60866558 | 6245,778029 | 0,000252812 |
| ENSMUSG00000042448 | Hoxd1 | 4,172550203 | 18,03278354 | 3,09637E-05 |
| ENSMUSG00000079277 | Hoxd3 | 9,529908633 | 739,2449296 | 2,4149E-08 |
| ENSMUSG00000101174 | Hoxd4 | 9,561734555 | 755,7339135 | 2,4149E-08 |
| ENSMUSG00000027102 | Hoxd8 | 5,677855052 | 51,19230467 | 1,43054E-07 |
| ENSMUSG00000043342 | Hoxd9 | 11,09493637 | 2187,301486 | 0,002738252 |
| ENSMUSG00000042745 | Id1 | 0,677773235 | 1,599668799 | 1,84246E-05 |
| ENSMUSG00000007872 | Id3 | 0,520324272 | 1,434277592 | 5,0012E-05 |
| ENSMUSG00000021379 | Id4 | -1,585449363 | 0,333220863 | 0,004221621 |
| ENSMUSG00000044030 | Irf2bp1 | 0,515493863 | 1,429483397 | 0,013744264 |
| ENSMUSG00000001504 | Irx2 | -0,491398465 | 0,711335236 | 0,033087944 |
| ENSMUSG00000031734 | Irx3 | 0,446526424 | 1,362755199 | 0,045880739 |
| ENSMUSG00000052837 | Junb | 1,380225355 | 2,603090293 | 0,021631405 |
| ENSMUSG00000074622 | Mafb | 0,761645839 | 1,695423676 | 0,027474686 |
| ENSMUSG00000059401 | Mamld1 | -0,712933861 | 0,610078223 | 0,042750452 |
| ENSMUSG00000002968 | Med25 | 0,408707653 | 1,327496128 | 0,031082887 |
| ENSMUSG00000001493 | Meox1 | 1,805943048 | 3,496576447 | 1,6132E-05 |
| ENSMUSG00000028496 | Mllt3 | 0,402006202 | 1,321344086 | 0,006790613 |
| ENSMUSG00000001566 | Mnx1 | 9,864929022 | 932,4800319 | 0,012183762 |
| ENSMUSG00000048450 | Msx1 | 0,737373104 | 1,667137507 | 5,86412E-10 |
| ENSMUSG00000019982 | Myb | -0,556908914 | 0,679757037 | 0,041178985 |
| ENSMUSG00000001496 | Nkx2-1 | -1,647400328 | 0,31921485 | 1,94283E-16 |
| ENSMUSG00000060601 | Nr1h2 | 0,431116608 | 1,348276704 | 0,020195894 |
| ENSMUSG00000019803 | Nr2e1 | -0,976463776 | 0,508223934 | 4,18687E-05 |
| ENSMUSG00000005917 | Otx1 | -0,748943003 | 0,595039356 | 0,014350143 |
| ENSMUSG00000026976 | Pax8 | 0,785121401 | 1,723237321 | 0,01201795 |
| ENSMUSG00000079466 | Prdm12 | 0,778721529 | 1,715609879 | 1,23654E-05 |
| ENSMUSG00000040478 | Prdm13 | 1,659339784 | 3,158719402 | 0,005269947 |
| ENSMUSG00000069378 | Prdm6 | 1,249883107 | 2,37822153 | 0,025449507 |
| ENSMUSG00000035456 | Prdm8 | 1,995165045 | 3,986617096 | 0,000581782 |
| ENSMUSG00000030350 | Prmt8 | -0,548593955 | 0,683686122 | 0,03745545 |
| ENSMUSG00000037992 | Rara | 0,711273358 | 1,637248556 | 1,84246E-05 |
| ENSMUSG00000032238 | Rora | -0,589222431 | 0,664701064 | 0,035970981 |
| ENSMUSG00000031665 | Sall1 | 0,52143492 | 1,435382185 | 0,004144219 |
| ENSMUSG00000024134 | Six2 | -0,739827981 | 0,598810747 | 0,001913767 |
| ENSMUSG00000038805 | Six3 | -1,188852206 | 0,438651709 | 4,32141E-09 |
| ENSMUSG00000021099 | Six6 | -1,129110124 | 0,457197645 | 0,000186874 |
| ENSMUSG00000036867 | Smad6 | 0,7662621 | 1,700857293 | 0,001587941 |
| ENSMUSG00000033006 | Sox10 | 1,228794754 | 2,343711114 | 3,65503E-12 |
| ENSMUSG00000046470 | Sox18 | 0,590005198 | 1,50525217 | 0,013790516 |
| ENSMUSG00000041540 | Sox5 | -0,418578111 | 0,748161633 | 0,014868248 |
| ENSMUSG00000075304 | Sp5 | 0,877387339 | 1,837045474 | 0,002701217 |
| ENSMUSG00000062327 | T | 1,590384987 | 3,011296961 | 0,014841756 |
| ENSMUSG00000028417 | Tal2 | -0,978835742 | 0,507389039 | 9,0666E-06 |
| ENSMUSG00000009097 | Tbx1 | 1,000791139 | 2,001097052 | 0,00209663 |
| ENSMUSG00000031965 | Tbx20 | -0,8942485 | 0,538027381 | 0,002252535 |
| ENSMUSG00000018263 | Tbx5 | -3,876687878 | 0,06807704 | 0,015076778 |
| ENSMUSG00000000782 | Tcf7 | 0,477198575 | 1,392037981 | 2,8892E-05 |
| ENSMUSG00000024985 | Tcf7l2 | -0,625428326 | 0,648227295 | 0,001636439 |
| ENSMUSG00000029723 | Tsc22d4 | 0,523864833 | 1,437801818 | 0,009716022 |
| ENSMUSG00000046982 | Tshz1 | 1,077680444 | 2,110639877 | 2,57736E-11 |
| ENSMUSG00000021217 | Tshz3 | 1,27122984 | 2,413672338 | 5,78232E-10 |
| ENSMUSG00000006270 | Vax1 | -2,591842688 | 0,165873729 | 6,06681E-06 |
| ENSMUSG00000049641 | Vgll2 | 1,177931597 | 2,262521649 | 0,021950461 |
| ENSMUSG00000021239 | Vsx2 | -2,691789102 | 0,15477141 | 5,86412E-10 |
| ENSMUSG00000016458 | Wt1 | 2,749034811 | 6,722672226 | 0,020817396 |
| ENSMUSG00000022708 | Zbtb20 | -0,566470365 | 0,675266848 | 0,032333146 |
| ENSMUSG00000021127 | Zfp36l1 | 0,603669105 | 1,519576289 | 0,034551371 |
| ENSMUSG00000039081 | Zfp503 | 0,954648265 | 1,938107057 | 4,22028E-15 |
| ENSMUSG00000085795 | Zfp703 | 0,464539567 | 1,379876898 | 0,000581782 |
| ENSMUSG00000054716 | Zfp771 | 0,6321967 | 1,549923167 | 0,013351283 |
| ENSMUSG00000053390 | Zfp952 | -0,460087396 | 0,726942221 | 0,007032758 |
