# Supplementary material for: Severe head dysgenesis resulting from imbalance between anterior and posterior ontogenetic programs
Source: Cell Death Dis. 2019 Oct 24;10(11):812. doi: 10.1038/s41419-019-2040-0 (PMC6813351; doi:10.1038/s41419-019-2040-0)
Supplement: Supplementary file 3 — Supplementary Figure Legend [file 41419_2019_2040_MOESM3_ESM.docx]

**Figure S1**

Morphology of one control and all mutant embryos of one littermate at E12.5 (**A**) and one littermate at E15.5 (**B**). Bars: 1 mm. In this figure, the pictures of the control and of one mutant embryo shown at each developmental stage are reproduced from the Figure 1C.
