# Supplementary figures and images for: Severe head dysgenesis resulting from imbalance between anterior and posterior ontogenetic programs
Source: Cell Death Dis. 2019 Oct 24;10(11):812. doi: 10.1038/s41419-019-2040-0 (PMC6813351; doi:10.1038/s41419-019-2040-0)

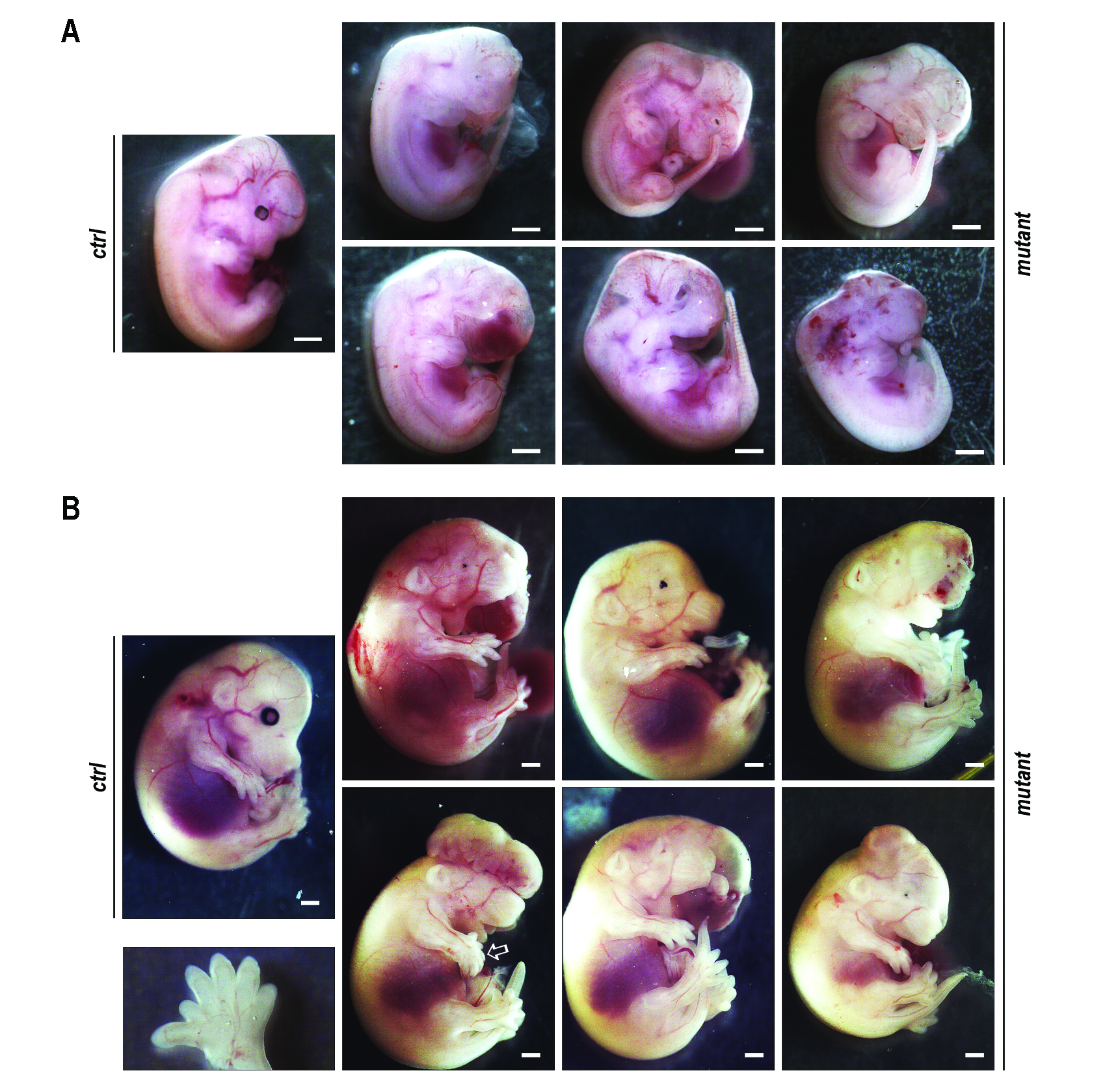

Supplement: Supplementary file 4 — Supplementary Fig S1 [file 41419_2019_2040_MOESM4_ESM.tif]
