# Supplementary material for: Severe head dysgenesis resulting from imbalance between anterior and posterior ontogenetic programs
Source: Cell Death Dis. 2019 Oct 24;10(11):812. doi: 10.1038/s41419-019-2040-0 (PMC6813351; doi:10.1038/s41419-019-2040-0)
Supplement: Supplementary file 5 — Declaration of contributions to article [file 41419_2019_2040_MOESM5_ESM.pdf]

DECLARATION OF CONTRIBUTIONS TO ARTICLE

ADMC

Manuscript Number: CDDIS-19-2593R

Journal Name: *Cell Death & Differentiation* (the 'Journal')

Proposed Title of the Contribution:

Severe head dysgenesis resulting from imbalance between anterior and posterior ontogenetic programs (the 'Contribution)

Author(s):

Emmanuelle Grall, Victor Gourain, Asmaa Nair,Elisabeth Martin, Marie-Christine Birling, Jean-Noël Freund, Isabelle Duluc (the 'Authors')

For all *CDD* articles, each person named as an author in the published version must be able to show he or she has contributed substantially to the article.

Authorship credit should be based on 1) substantial contributions to conception and design, acquisition of data, or analysis and interpretation of data; 2) drafting the article or revising it critically for important intellectual content; and 3) final approval of the version to be published. Authors should meet conditions 1, 2 and 3.

Any person who cannot be shown to have made a substantial contribution to the article cannot be listed as an author in the final version. The name of any person who is deemed to have made a minor contribution can, however, appear in the Acknowledgments section of the article.

Please complete the table below to indicate the contributions of all named authors to the manuscript.

Author Full Name:	Specification of Contribution to the Manuscript:
Emmanuelle Grall	contributed to phenotype analyses
Victor Gourain	performed RNAseq analysis and contributed to the interpretation of the data
Asmaa Nair	contributed to phenotype analyses
Elisabeth Martin	contributed to phenotype analyses
Marie-Christine Birling	developed the RsCDX2 mouse
Jean-Noël Freund	conceived and designed the study, contributed to data acquisition, analysis and interpretation, and wrote the manuscript
Isabelle Duluc	conceived and designed the study, developed the RsCDX2 mouse, contributed to data acquisition, analysis and interpretation, and wrote the manuscript

Please complete the table below to indicate the contributions of all named authors to the figures.

Figure 1:

Emmanuelle Grall
Asmaa Nair
Elisabeth Martin
Marie-Christine Birling
Jean-Noël Freund
Isabelle Duluc

Figure 2:

Victor Gourain
Jean-Noël Freund
Isabelle Duluc

Figure 3:

Victor Gourain
Jean-Noël Freund
Isabelle Duluc

Figure 4:

Emmanuelle Grall
Elisabeth Martin
Jean-Noël Freund
Isabelle Duluc

Figure 5:

Elisabeth Martin
Jean-Noël Freund
Isabelle Duluc

Figure 6:

Elisabeth Martin
Jean-Noël Freund
Isabelle Duluc

Signed for and on behalf of the Author(s):	Print Name:	Date:
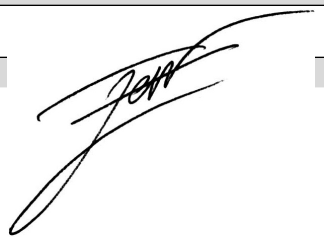	Jean-Noel FREUND	Octobre 1st, 2019
